# Advanced Maternal Age‐associated SIRT1 Deficiency Compromises Trophoblast Epithelial−Mesenchymal Transition through an Increase in Vimentin Acetylation

**DOI:** 10.1111/acel.13491

**Published:** 2021-10-03

**Authors:** Liling Xiong, Xuan Ye, Zhi Chen, Huijia Fu, Sisi Li, Ping Xu, Jiaxiao Yu, Li Wen, Rufei Gao, Yong Fu, Hongbo Qi, Mark D. Kilby, Richard Saffery, Philip N. Baker, Chao Tong

**Affiliations:** ^1^ Department of Obstetrics The First Affiliated Hospital of Chongqing Medical University Chongqing China; ^2^ Ministry of Education‐International Collaborative Laboratory of Reproduction and Development Chongqing China; ^3^ State Key Laboratory of Maternal and Fetal Medicine of Chongqing Municipality Chongqing China; ^4^ Laboratory of Reproductive Biology School of Public Health and Management Chongqing Medical University Chongqing China; ^5^ Institute of Metabolism and System Research University of Birmingham Edgbaston UK; ^6^ Cancer, Disease and Developmental Epigenetics Murdoch Children’s Research Institute Parkville Victoria Australia; ^7^ College of Life Sciences University of Leicester Leicester UK

**Keywords:** advanced maternal age, epithelial−mesenchymal transition, placenta, senescence, SIRT1, trophoblast

## Abstract

Advanced maternal age (AMA) pregnancies are rapidly increasing and are associated with aberrant trophoblast cell function, poor placentation, and unfavorable pregnancy outcomes, presumably due to premature placental senescence. SIRT1 is an NAD^+^‐dependent deacetylase with well‐known antiaging effects, but its connection with placental senescence is unreported. In this study, human term placentas and first‐trimester villi were collected from AMA and normal pregnancies, and a mouse AMA model was established by cross breeding young and aged male and female C57 mice. *SIRT1* expression and activity in HTR8/SVneo cells were genetically or pharmacologically manipulated. Trophoblast‐specific *Sirt1*‐knockout (KO) mouse placentas were generated by mating *Elf5*‐Cre and *Sirt1* ^fl/fl^ mice. Trophoblast cell mobility was assessed with transwell invasion and wound‐healing assays. SIRT1‐binding proteins in HTR8/SVneo cells and human placental tissue were identified by mass spectrometry. We identified SIRT1 as the only differentially expressed sirtuin between AMA and normal placentas. It is downregulated in AMA placentas early in the placental life cycle and is barely impacted by paternal age. SIRT1 loss upregulates P53 acetylation and P21 expression and impairs trophoblast invasion and migration. *Sirt1*‐KO mouse placentas exhibit senescence markers and morphological disruption, along with decreased fetal weight. In trophoblasts, SIRT1 interacts with vimentin, regulating its acetylation. In conclusion, SIRT1 promotes trophoblast epithelial−mesenchymal transition (EMT) to enhance invasiveness by modulating vimentin acetylation. AMA placentas are associated with premature senescence during placentation due to SIRT1 loss. Therefore, SIRT1 may be an antiaging therapeutic target for improving placental development and perinatal outcomes in AMA pregnancies.

## Graphical abstract

Both the first‐trimester villi and full‐term placentas from pregnancies of advanced maternal age (AMA) are associated with the significant downregulation of SIRT1 and accumulation of P53, P21, and β‐galactosidase in trophoblasts, where SIRT1 directly interacts with vimentin and modulates the acetylation status of vimentin, thus affecting the EMT process and ultimately influencing the invasiveness and mobility of trophoblasts. Both P53–P21 axis‐dependent cellular senescence and vimentin acetylation‐induced EMT suppression may orchestrate premature placental senescence and eventually lead to a variety of adverse pregnancy outcomes.

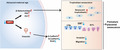

## 1 INTRODUCTION

Maternal age has increased steadily over the past decades; specifically, the number of births to women in their early 40s rose by 2% from 2017 to 2018 in the UK, and the rate has risen almost continuously since 1985 (Martin et al., 2019). Very similar trends have also been observed in North America and elsewhere (Abel et al., 2002). This phenomenon is due mainly to social and cultural factors and advances in assisted reproductive technologies (Cooke & Davidge, 2019). Advanced maternal age (AMA) is usually defined as maternal age ≥35 years (de Jongh et al., 2015), and cumulative evidence suggests that AMA is associated with various adverse pregnancy outcomes, such as fetal growth restriction (FGR), intrauterine fetal death, preeclampsia (PE), gestational diabetes mellitus (GDM), and preterm birth (Abel et al., 2002; Andersen et al., 2000; du Fosse et al., 2020; Miremerg et al., 2020; Salem Yaniv et al., 2011). A recent study reported that AMA embryos demonstrate elevations in well‐established biomarkers of aging, including the tumor suppressor P53, the cell cycle regulator P21, and the senescence‐associated secretory phenotype‐associated marker IL6 (Baker et al., 2011; Cha & Aronoff, 2017; Kawagoe et al., 2020). Given that the placenta develops from the trophectoderm of the embryo, we speculate that the AMA placenta may also be associated with premature senescence.

Placental senescence can be induced by various forms of cellular stress, including oncogene activation, telomere shortening, oxidative stress, and other types of stress (Vigneron & Vousden, 2012). Pathological examinations of placentas from women with AMA have revealed higher frequencies of fetal vascular malperfusion, villous maturation, and chorangiosis (Torous & Roberts, 2020). Specifically, the trophoblast‐derived portions of the placentas of aged female mice are drastically reduced in size (Woods et al., 2017).

Sirtuins are class III histone deacetylases that have been shown to influence diverse biological events, such as senescence, energy metabolism, and apoptosis (Giblin et al., 2014; Haigis & Sinclair, 2010). To date, seven sirtuin family members (SIRT1–7) have been identified, and these enzymes are conserved throughout evolution from yeast to humans (Brooks & Gu, 2009). The expression of sirtuins is spatially different; SIRT1, SIRT6, and SIRT7 are localized principally to the nucleus, SIRT2 exists mainly in the cytosol, and SIRT3, SIRT4, and SIRT5 are expressed in mitochondria (Michishita et al., 2005). Limited evidence shows that SIRT1 is anti‐inflammatory in trophoblasts of the human placenta (Lappas et al., 2011); however, SIRT2 is reported to be restricted to syncytiotrophoblasts, and its downregulation may induce necroptosis in the PE placenta (Hannan et al., 2017). Although these observations suggest potential crucial roles for sirtuins in the placenta, their roles in placental senescence remain largely unknown.

The placenta is a temporary organ that links the fetus to the maternal uterus. During early pregnancy, cytotrophoblasts (CTBs) derived from the trophectoderm of the blastocyst after implantation function as precursor cells of the human placenta and form the placental villous structure. Villous CTBs undergo partial epithelial−mesenchymal transition (EMT) and differentiate into extravillous trophoblasts (EVTs) to acquire invasiveness to infiltrate the maternal decidua (DaSilva‐Arnold et al., 2015; Davies et al., 2016). Our study and other previous studies have clearly demonstrated the importance of EMT in trophoblast function and placental development (Chen et al., 2018; Davies et al., 2016; Owusu‐Akyaw et al., 2019). The EMT process is characterized by the breakdown of cell−cell adhesions, loss of epithelial phenotypes, and cell depolarization, accompanied by downregulation of E‐cadherin and increased expression of mesenchymal markers, such as vimentin and N‐cadherin (Bai et al., 2018; DaSilva‐Arnold et al., 2019). Vimentin is one of the most conserved and abundant proteins involved in the EMT process and undergoes various important posttranslational modifications (PTMs), such as phosphorylation, glycosylation, sumoylation, and acetylation (Snider & Omary, 2014). However, researchers have not clearly determined whether trophoblastic sirtuin‐mediated acetylation of vimentin is involved in the EMT process. In the present study, we aimed to explore the role of sirtuins in AMA‐associated placental senescence, as well as the underlying mechanism that involves EMT.

## 2 RESULTS

### 2.1 The AMA term placenta is associated with senescence and SIRT1 deficiency

**FIGURE 1:**
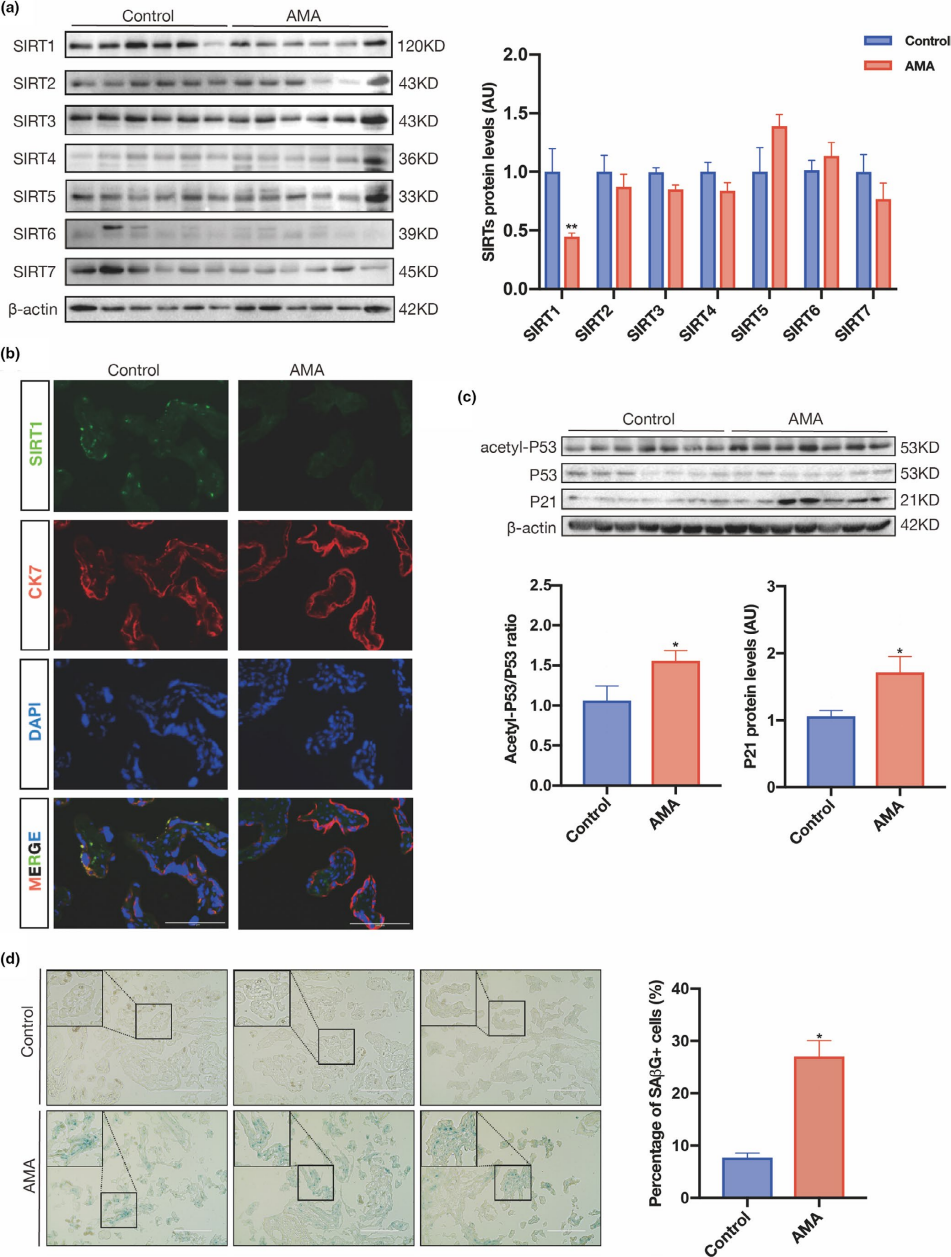
SIRT1 is downregulated in AMA term placenta and is associated with placental senescence. (a) Western blot analysis of the expression of SIRT1–7 in human term placental tissues (n = 6 in the control group and AMA group). (b) IF staining of SIRT1 (green) and CK7 (red) in frozen sections of human term placentas; nuclei were counterstained with DAPI (blue). Scale bar: 100 μm. (c) Western blot analysis of the levels of acetyl‐P53, total P53, and P21 in human term placentas (n = 6 in the control group and AMA group). (d) SAβG staining of human term placental sections. Scale bar: 200 μm. AU, arbitrary unit. Data were analyzed using Student's t‐test, *p < 0.05. All data are presented as the mean ± SEM. All experiments were performed in triplicate

First, we determined the expression patterns of SIRT1–7 in human term placenta. The results showed that SIRT1 was the only sirtuin that was significantly downregulated in the AMA group compared with the control group and that the levels of the other sirtuins were unchanged (Figure 1a). This downregulation of SIRT1 in the AMA placenta was then confirmed by immunofluorescence (IF) staining, which also demonstrated that SIRT1 was located primarily in trophoblasts (Figure 1b). Since SIRT1 activity is driven by NAD^+^, we then measured NAD^+^ and NADH in human term placentas, and the results showed that the NAD^+^/NADH ratio did not differ between the control and AMA groups (Figure S1a). Since the SIRT1−P53 axis plays an important role in modulating pathways that are involved in tissue homeostasis and senescence, SIRT1‐mediated acetylation of P53 reduces the expression of its downstream target P21 (Lee & Gu, 2013; Ong & Ramasamy, 2018). Therefore, we next examined P53 and P21 in human placentas. Western blotting demonstrated that the levels of acetylation of the P53 and P21 proteins were significantly increased in the AMA placenta (Figure 1c). RT‐qPCR showed that *P21* mRNA levels were upregulated in AMA term placenta but that *P53* levels were not significantly different between the two groups (Figure S2a). These facts suggest that SIRT1 deficiency in the placenta may promote *P21* transcription due to the acetylation of P53. In addition, more senescence‐associated β‐galactosidase (SaβG)‐positive cells were observed in the AMA term placenta than in the control placenta (Figure 1d), confirming that the AMA placenta is associated with increased cellular senescence, which might be attributed to the loss of SIRT1.

### 2.2 Placental SIRT1 decreases in the AMA placenta beginning in early pregnancy

**FIGURE 2:**
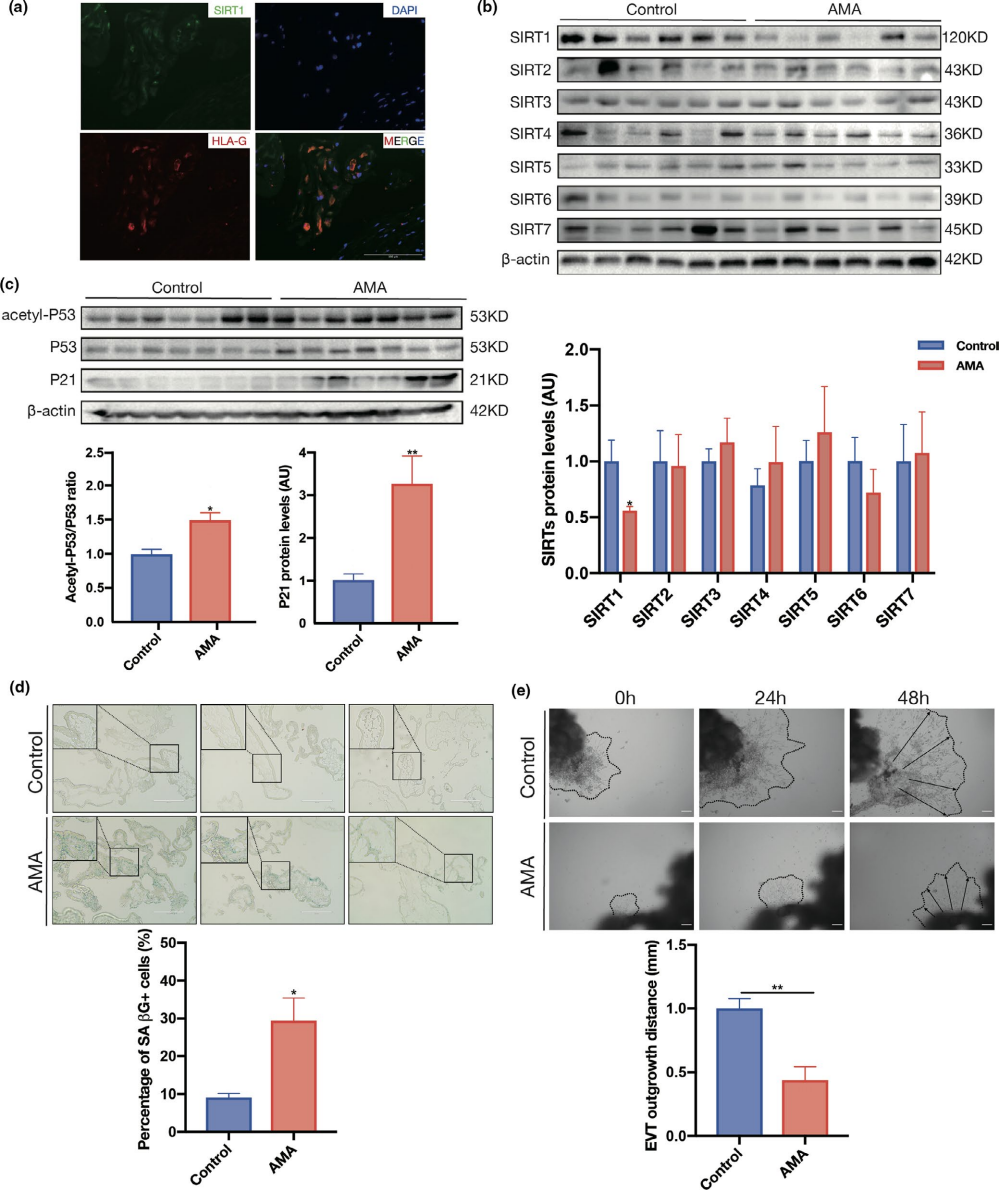
SIRT1 decreases in early AMA pregnancy and is associated with premature placental senescence. (a) IF detection of SIRT1 expression in first‐trimester human decidua. IF staining with an antibody against SIRT1 is shown in green, HLA‐G staining is shown in red, and nuclei are shown in blue (DAPI staining). (b) The expression of SIRT1–7 in human first‐trimester villi was determined using Western blotting (n = 6 in the control group and AMA group). (c) Western blot analysis of the levels of acetyl‐P53, P53, and P21 in human first‐trimester villi (n = 6 in the control group and AMA group). (d) SAβG staining of human first‐trimester villi sections. Scale bar: 200 μm. (e) Migration (dotted line circled area) of EVTs induced from human first‐trimester villous explants. n = 3; scale bar: 200 μm; AU, arbitrary unit. Data were analyzed using Student's t‐test, *p < 0.05, **p < 0.01. All data are presented as the mean ± SEM. All experiments were performed in triplicate

Next, we detected SIRT1 expression and localization in the maternal decidua using IF staining. IF staining revealed strong SIRT1 expression in EVT cells in the first‐trimester decidua, as evidenced by costaining with human leukocyte antigen G (HLA‐G) (Figure 2a). It is widely believed that the placenta progressively ages to term, as shown by a decline in morphological and physiological senescence. Therefore, although we observed downregulation of SIRT1 and more senescence biomarkers in the AMA term placenta, it is still unclear whether these are consequent manifestations of accelerated aging during gestation or intrinsic distinctions between AMA and non‐AMA pregnancies. We investigated the expression patterns of SIRT1 in human first‐trimester villi and full‐term placentas collected from control and AMA pregnancies to clarify this issue. Our data showed that placental SIRT1 protein expression peaked in the first trimester and gradually decreased as gestational age increased (Figure S3). We then evaluated the protein expression of sirtuins in human first‐trimester villi and found that SIRT1 was decreased in the AMA group compared with the control group, but that there were no differences in the other sirtuins between the two groups (Figure 2b). In accordance with the change in SIRT1, the acetyl‐P53‐to‐P53 ratio and P21 protein level were both elevated in the first‐trimester villi of AMA pregnancies (Figure 2c). Further validation by RT‐qPCR confirmed that the mRNA levels of *P53* and *P21* were upregulated in AMA villi (Figure S2b). Consistently, many more SAβG‐positive cells were found in the first‐trimester villi from the AMA group than in those from the control group (Figure 2d). This evidence indicates that the lower SIRT1 level observed in the AMA term placenta results mainly from SIRT1 deficiency beginning at placentation rather than accelerated loss during pregnancy.

In addition, because first‐trimester villi aggressively invade and migrate to expand the area of the placenta, we evaluated the outgrowth capability of villous explants collected from normal and AMA pregnancies ex vivo. The results showed that the outgrowth distance of villous explants within 48 h in the AMA group was nearly 60% shorter than that in the control group (Figure 2e). Taken together, these results show that the AMA placenta has SIRT1 deficiency beginning at the early stage of pregnancy, which may contribute to premature senescence and maldevelopment.

### 2.3 The mouse AMA placenta is associated with downregulation of Sirt1 and senescence

**FIGURE 3:**
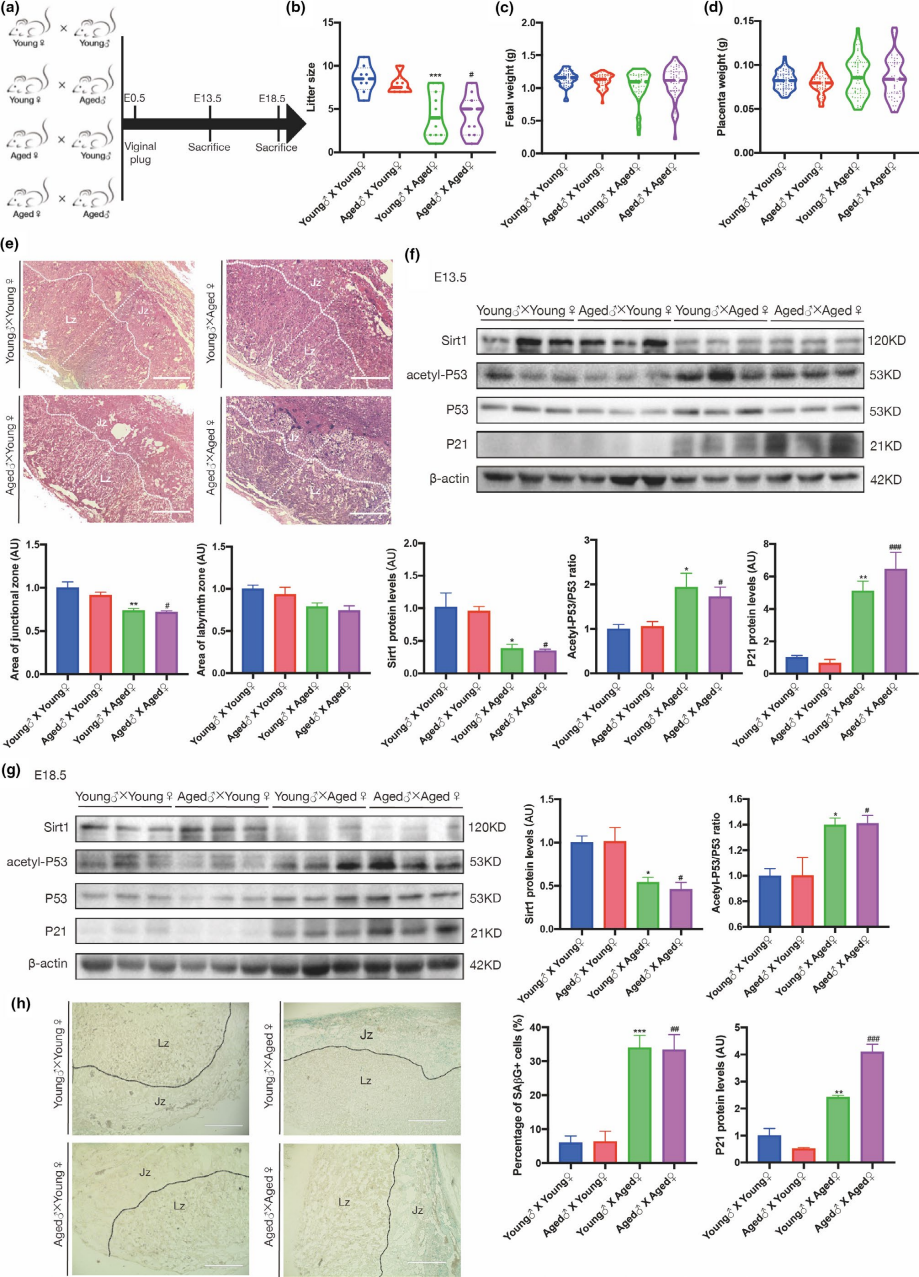
Advanced maternal age is associated with adverse birth outcomes and downregulation of SIRT1 in the mouse placenta. (a) The mating plan for the animals. (b) Average litter size. (c) Birth weight of viable fetuses. (d) Placental weight of viable fetuses among different mating groups. (e) H&E staining of sections from E13.5 placentas from different mating groups. Jz, junctional zone; Lz, labyrinth zone. Scale bar: 400 μm. Western blot analysis of the levels of SIRT1, acetyl‐P53, P53, and P21 in (f) E13.5 and (g) E18.5 mouse placentas from different mating groups (n = 3 biologically independent animals from each group). (h) SAβG staining of E13.5 mouse placental sections from different mating groups. Jz, junctional zone; Lz, labyrinth zone. Scale bar: 400 μm. AU, arbitrary unit. Data were analyzed using one‐way ANOVA, followed by Tukey's or Sidak's multiple comparison tests, n = 6; *p < 0.05; **p < 0.01; ***p < 0.001 vs. Young♂ x Young♀ group; #*p* < 0.05; ##*p* < 0.01; ###*p* < 0.001 vs. Aged♂ x Young♀ group. All data are presented as the mean ± SEM. All experiments were performed in triplicate

As a temporal organ that exists only during gestation, the placenta develops from the trophectoderm of the embryo. Therefore, theoretically, it can be influenced by both maternal and paternal factors, including age. To assess the contribution of parental age to the basal status of placental Sirt1, aged and young female mice were bred with aged or young males, as depicted in Figure 3a.

The litter sizes of young females were comparable regardless of male age; however, aged females had significantly smaller litter sizes, which were not further compromised in aged breeding pairs (Figure 3b). However, neither the average fetal weight nor the average placental weight of viable fetuses differed among the groups (Figure 3c,d); this finding is consistent with previous reports (Woods et al., 2017). Nonetheless, the distribution of fetal weight and placental weight were increased significantly in the aged group. Morphological assessments of the uteroplacental units revealed a marked reduction in the junctional zone (Jz) area in aged females (Figure 3e). These results may imply that aged dams have an impaired capability of carrying conceptuses, possibly due to maldevelopment of placentas, and therefore adapt by reducing the litter size to allow a sufficient nutrient supply and ensure the survival of the offspring.

To validate whether the placental Sirt1 level in this mouse model mimics that in the human AMA placenta, mouse placentas from the aforementioned four groups were harvested on both embryonic days (E) 13.5 and E18.5. Compared with that in young dams, the SIRT1 level in the placenta collected on E13.5 was lower in aged females, and the acetyl‐P53 to total P53 ratio and P21 level were increased; notably, the age of the male did not alter these discrepancies between young and aged female placentas (Figure 3f). Similar results were also obtained for mouse term placentas collected on E18.5 (Figure 3j). However, the NAD^+^/NADH level in the mouse placenta did not differ between young and aged females (Figure S1b). Consistently, a large number of SAβG‐positive cells had accumulated in the placentas collected from the two groups of aged females on E13.5 (Figure 3h) and E18.5 (Figure S4). These facts confirmed that placental deficiency of Sirt1 emerges at the early stage of the placental life cycle and is likely due to maternal rather than paternal age.

### 2.4 SIRT1 deficiency induces senescence and impairs trophoblast migration and invasion

**FIGURE 4:**
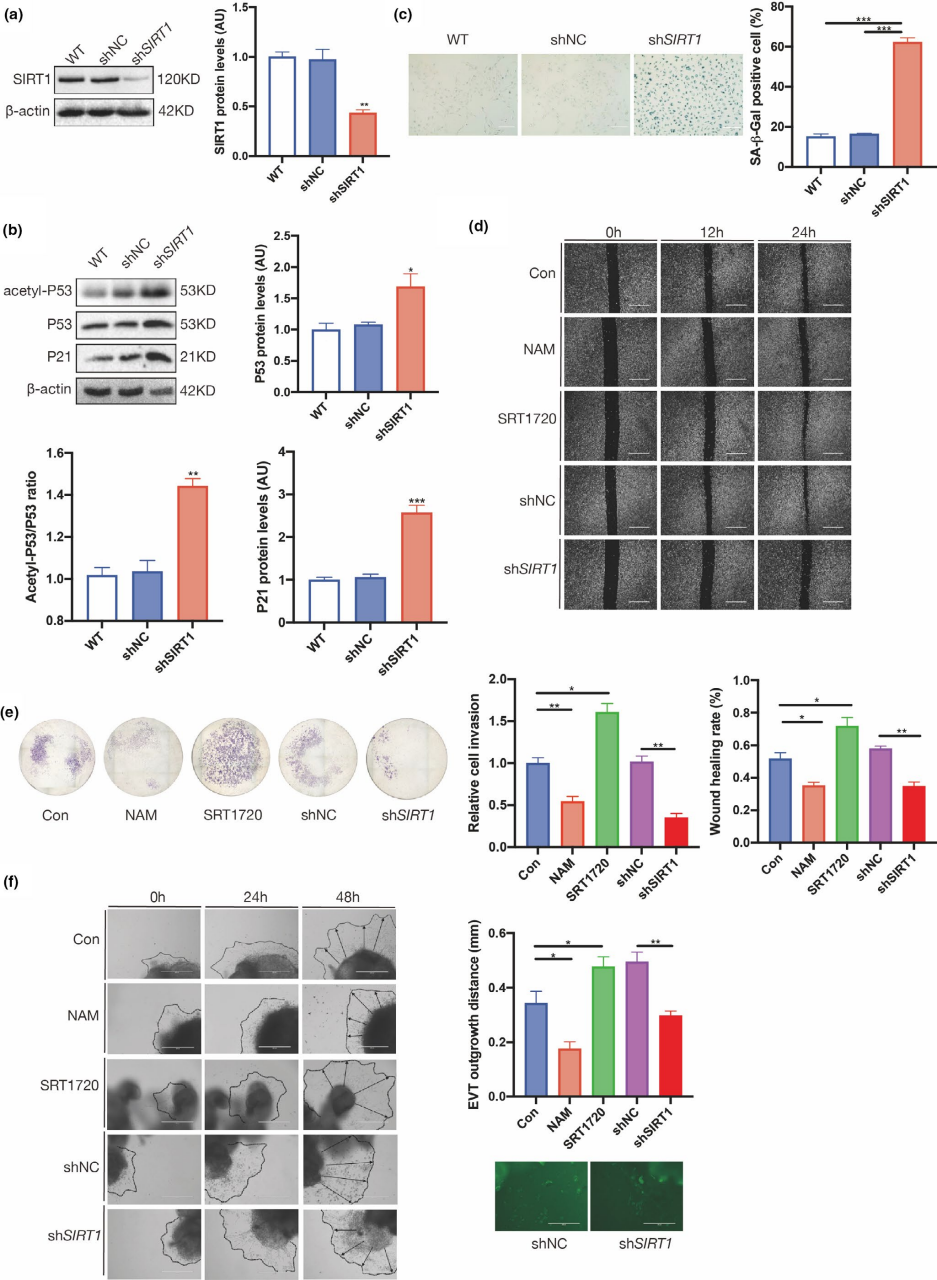
SIRT1 deficiency induces senescence and impairs the migration and invasion of trophoblast cell lines. (a) Western blot analysis of SIRT1 expression in HTR8/SVneo cells. (b) Western blot analysis of acetyl‐P53, P53, and P21 in HTR8/SVneo cells. (c) SAβG staining of HTR8/SVneo cells. Scale bar: 200 μm. (d) Wound‐healing assay; scale bar: 100 μm. (e) Transwell assay of HTR8/SVneo cells treated with SRT1720 or nicotinamide (NAM) and transfected with sh*SIRT1*. (f) Migration (dotted line circled area) of EVTs induced from human first‐trimester villous explants treated with sh*SIRT1*, SRT1720 and NAM. Scale bar: 200 μm. Data were analyzed using one‐way ANOVA; *p < 0.05, **p < 0.01, ***p < 0.001. All data are presented as the mean ± SEM. All experiments were performed in triplicate

As a temporal organ connecting the embryo to the mother during gestation, the placenta develops from the trophectoderm and mainly consists of trophoblasts. To ascertain whether loss of SIRT1 causes trophoblast senescence, we established a Sirt1‐deficient HTR8/SVneo trophoblast cell line, which had a nearly 60% decrease in SIRT1 protein expression compared with control cells (Figure 4a). As a result, the mRNA level of *P21* but not *P53* in sh*SIRT1* cells was significantly elevated (Figure S2c), while both the P53 and P21 protein levels were largely increased; notably, the acetylation of P53 was dramatically enhanced (Figure 4b). In line with these observations, SAβG‐positive staining was more than 3‐fold higher in shSIRT1 cells (Figure 4c).

Furthermore, to assess the effects of SIRT1 on trophoblast functions, HTR8/SVneo cells in which *SIRT1* expression or SIRT1 activity was manipulated were subjected to migration and invasion assays. As shown in the wound‐healing assay, the migratory capability was markedly suppressed in sh*SIRT1* cells and wild‐type (WT) cells treated with the SIRT1 inhibitor nicotinamide (NAM). In contrast, treatment with the SIRT1‐specific activator SRT1720 significantly improved the migration of WT cells (Figure 4d). Consistently, the results of the Matrigel transwell assay revealed that ablation or inhibition of SIRT1 led to a significant loss of invasiveness among HTR8/SVneo cells, while activation of SIRT1 by SRT1720 remarkably increased invasion (Figure 4e). The same treatments were applied to human first‐trimester villous explants. Similarly, the outgrowth of villous explants within 48 h was significantly promoted by SRT1720 treatment but inhibited by NAM treatment or sh*SIRT1* (Figure 4f). Taken together, these in vitro and ex vivo data indicate that SIRT1 deficiency in trophoblasts induces senescence and consequently compromises cellular functions.

### 2.5 Trophoblast‐specific *Sirt1* deletion induces placental senescence and maldevelopment in mice

**FIGURE 5:**
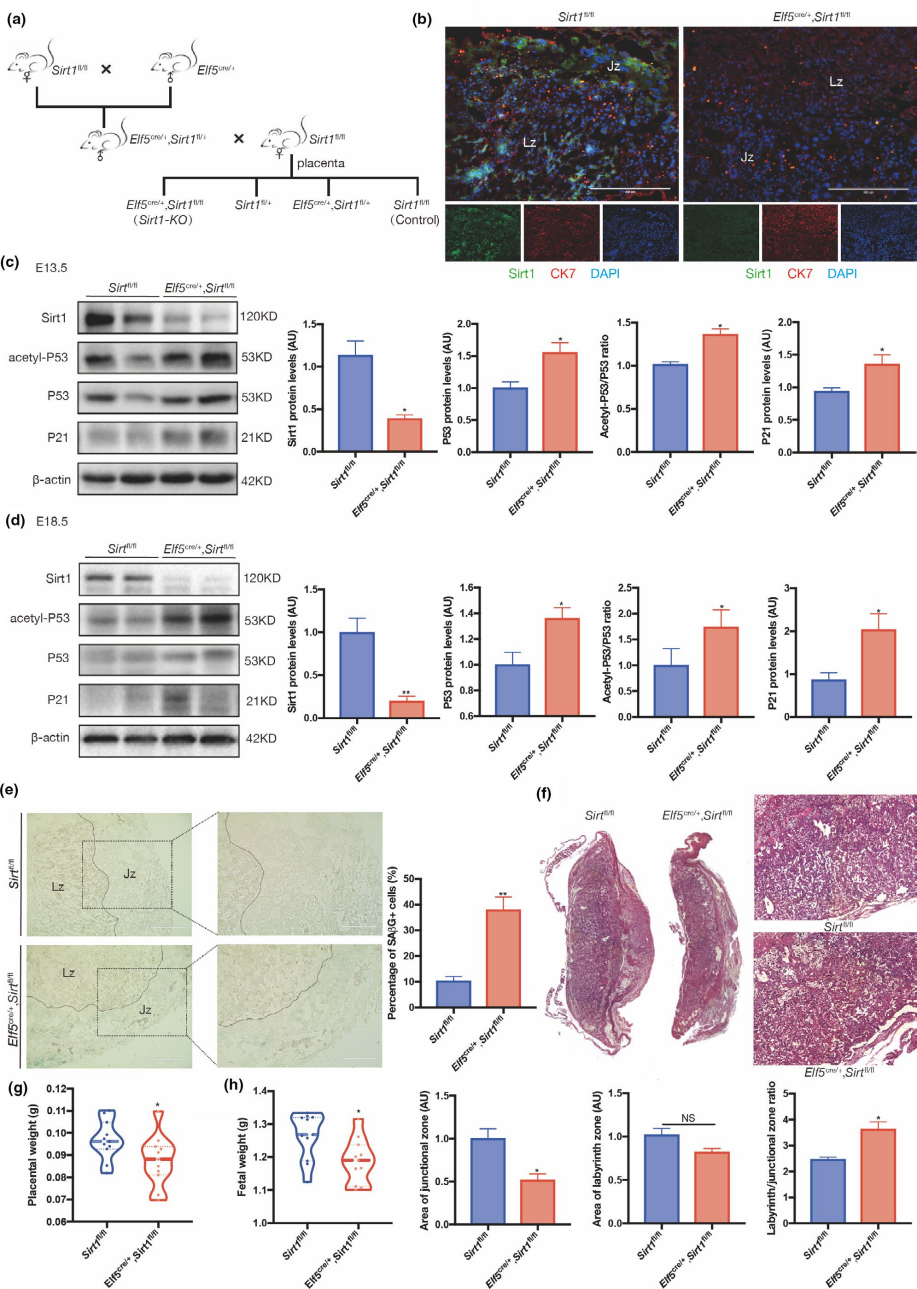
Senescence and maldevelopment in the *Sirt*1‐deficient mouse placenta. (a) The mating plan for generating *Sirt1*‐deficient mouse placentas. (b) IF staining of Sirt1 (green), CK7 (red) and DAPI (blue) in E13.5 *Elf5* ^cre/+^, *Sirt1* ^fl/fl^ and *Sirt1* ^fl/fl^ mouse placental sections. Western blot analysis of SIRT1, acetyl‐P53, P53, and P21 levels in (c) E13.5 and (d) E18.5 mouse placentas (n = 3 biologically independent animals from each group). (e) SAβG staining of E13.5 mouse placenta from *Elf5* ^cre/+^, *Sirt1* ^fl/fl^ and *Sirt1* ^fl/fl^ mice; scale bar: 400 μm. (f) H&E staining of sections from *Elf5* ^cre/+^, *Sirt1* ^fl/fl^ and *Sirt1* ^fl/fl^ placentas collected on E18.5. Weights of (g) the *Elf5* ^cre/+^, *Sirt1* ^fl/fl^ and *Sirt1* ^fl/fl^ placentas and (h) the corresponding fetus on E18.5 are shown; scale bars, 400 μm. AU, arbitrary unit. Data were analyzed using Student's t‐test, n = 6, *p < 0.05, **p < 0.01. All data are presented as the mean ± SEM. All experiments were performed in triplicate

Immunohistochemistry (IHC) was performed to ascertain the localization of SIRT1 in the mouse placenta, and Sirt1 was ubiquitously expressed in the junctional layer and labyrinthine layer of C57BL/6 mouse E18.5 placenta. Serial sections of mouse placenta stained with antibodies against cytokeratin 7 (CK7) and trophoblast‐specific protein alpha (Tpbpa) revealed that SIRT1 was mainly expressed in trophoblast cells (Figure S5). Therefore, adult female mice in which the *Sirt1* gene was conditionally deleted in placental trophoblast cells were generated by mating *Sirt1* floxed (*Sirt1* ^fl/fl^) females with *Elf5* ^cre/+^, *Sirt1* ^fl/+^ males to further explore the function of SIRT1 in vivo (Figure 5a). Sirt1‐deficient placentas were identified by the genotype *Elf5* ^cre/+^, *Sirt1* ^fl/fl^, while *Sirt1* ^fl/fl^ placentas were used as controls (Table S4 and Figure S6). The ablation of Sirt1 protein in *Elf5* ^cre/+^, *Sirt1* ^fl/fl^ mice was first confirmed by IF staining (Figure 5b), and western blotting indicated that compared with those in the placentas of *Sirt1* ^fl/fl^ littermates, in addition to increased acetylation of P53, the levels of both the P53 and P21 proteins were increased in *Elf5* ^cre/+^, *Sirt1* ^fl/fl^ placentas at E13.5 and E18.5 (Figure 5c,d). Furthermore, on E13.5, SAβG staining was enriched in the Jz and notably higher in *Elf5* ^cre/+^, *Sirt1* ^fl/fl^ placentas, as the proportion of SAβG‐positive cells was approximately 3‐fold greater than that in the *Sirt1* ^fl/fl^ placentas (Figure 5e). This result may have contributed to the disrupted labyrinth/Jz ratio in *Elf5* ^cre/+^, *Sirt1* ^fl/fl^ placentas, as the Jz area was significantly reduced while the labyrinth was barely compromised (Figure 5f). Hence, unsurprisingly, significantly lower placental and birth weights were observed among *Elf5* ^cre/+^, *Sirt1* ^fl/fl^ placentas (Figure 5g,h).

### 2.6 SIRT1 regulates trophoblast EMT by deacetylating vimentin

**FIGURE 6:**
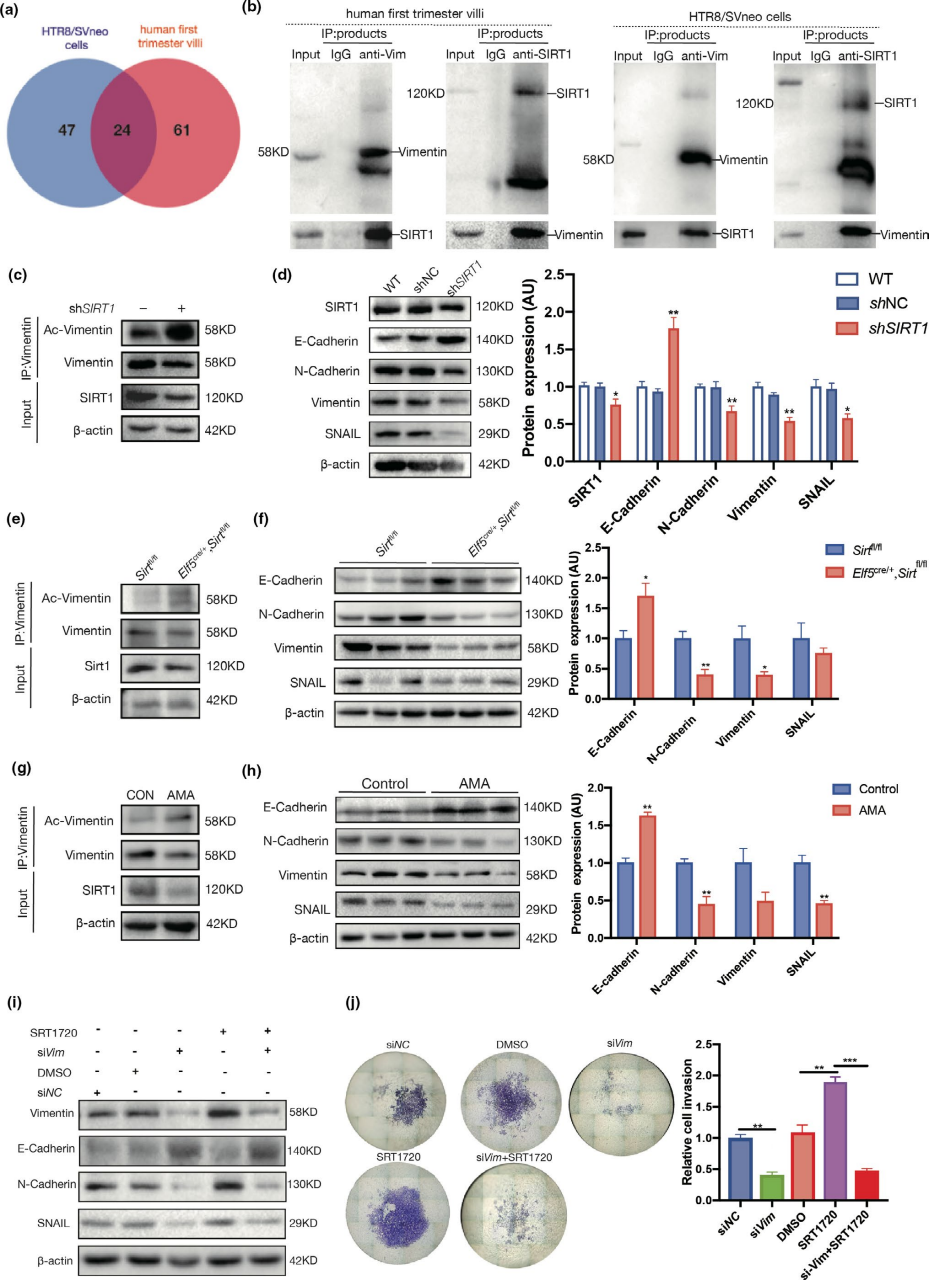
SIRT1 promotes trophoblast EMT. (a) Venn diagram of SIRT1‐binding proteins in HTR8/SVneo cells and human first‐trimester villi profiled by IP‐MS. (b) Reciprocal Co‐IP of SIRT1 and vimentin in human first‐trimester villi (left panel) and HTR8/SVneo cells (right panel). (c) Acetylation of vimentin in WT and sh*SIRT1* HTR8/SVneo cells. (d) Western blot analysis of E‐cadherin, N‐cadherin, vimentin, and SNAIL in WT, shNC, and sh*SIRT1* HTR8/SVneo cells. (e) Acetylation of vimentin in *Elf5* ^cre/+^, *Sirt1* ^fl/fl^ and *Sirt1* ^fl/fl^ mouse placentas collected on E13.5. (f) Western blot analysis of E‐cadherin, N‐cadherin, vimentin, and SNAIL levels in *Elf5* ^cre/+^, *Sirt1* ^fl/fl^ and *Sirt1* ^fl/fl^ mouse placentas collected on E13.5; n = 3. (g) Acetylation of vimentin in first‐trimester villi of AMA and control pregnancies. (h) Western blot analysis of E‐cadherin, N‐cadherin, vimentin, and SNAIL levels in first‐trimester villi from women with AMA and control women; n = 3. AU, arbitrary unit. Data were analyzed using Student's t‐test; *p < 0.05, **p < 0.01. All data are presented as the mean ± SEM. All experiments were performed in triplicate. (i) Transwell assays of Htr8/SVneo cells were performed after 48 h of treatment with si*Vim*, SRT1720 or both. (j) Western blot analysis of E‐cadherin, N‐cadherin, vimentin, and SNAIL levels in Htr8/SVneo cells treated with si*Vim*, SRT1720, or both for 48 h. Data were analyzed using one‐way ANOVA; **p < 0.01, ***p < 0.001. All data are expressed as the mean ± SEM

To characterize the mechanisms underlying the regulation of trophoblast invasion and placental development by SIRT1, we investigated the potential downstream target proteins in trophoblasts that were regulated by SIRT1 through modulation of acetylation. To this end, the immunoprecipitation (IP) products of SIRT1 in the lysate of human first‐trimester villous tissue and HTR8/SVneo cells were subjected to proteomics analyses by LC‐MS/MS. The results showed that 71 proteins were bound to SIRT1 in HTR8/SVneo cells, while 85 SIRT1‐binding proteins were identified in human villi (Tables S5 and S6). Twenty‐four proteins, including vimentin, interacted with SIRT1 in both human villi and HTR8/Svneo cells (Figure 6a). Vimentin has been reported to be acetylated at K120, and deacetylation of this site is associated with increased migration of hepatocellular carcinoma cells (Guo et al., 2018). Therefore, we first confirmed the protein−protein interaction between SIRT1 and vimentin in human villi and HTR8/Svneo cells by reciprocal Coimmunoprecipitation (Co‐IP) experiments (Figure 6b). To examine whether vimentin is the substrate of SIRT1‐catalyzed deacetylation in trophoblasts, we examined the impact of SIRT1 on the acetylation of vimentin in HTR8/Svneo cells. As expected, acetyl‐vimentin was substantially increased in the presence of sh*SIRT1*, while the total vimentin level was maintained (Figure 6c).

Vimentin is a well‐known biomarker of EMT, which is a fetal developmental process that drives metastasis of tumor cells (Das et al., 2019). Trophoblasts share a large number of similarities with cancer cells, including the acquisition of invasion properties. Therefore, we next evaluated the role of SIRT1 in modulating trophoblastic EMT. Our results showed that *SIRT1* knockdown increased the expression of E‐cadherin in HTR8/Svneo cells but suppressed that of N‐cadherin, vimentin, and SNAIL (Figure 6d). Similarly, acetylated vimentin levels were obviously increased in E13.5 *Elf5* ^cre/+^, *Sirt1* ^fl/fl^ mouse placentas (Figure 6e), along with elevations in E‐cadherin and reductions in N‐cadherin, vimentin, and SNAIL levels (Figure 6f). Finally, we assessed the acetylation of vimentin and EMT in human first‐trimester villi collected from control and AMA pregnancies and found that the acetylation of vimentin was enhanced in the AMA group (Figure 6g); moreover, the expression levels of N‐cadherin, vimentin, and SNAIL were decreased in AMA villi, while E‐cadherin was remarkably increased (Figure 6h).

Interference with vimentin expression was induced in HTR8/Svneo cells to study the role of vimentin in SIRT1‐dependent trophoblast EMT. Based on our results, downregulation of vimentin also decreased the levels of other EMT markers. More importantly, the loss of vimentin impeded the regulatory effects of SRT1720 on E‐cadherin, N‐cadherin, and SNAIL (Figure 6i). Consistent with these results, the stimulation of HTR8/Svneo cells with SRT1720 treatment was largely abolished in the presence of si*Vim* (Figure 6j). Taken together, these facts strongly suggest that vimentin may play a pivotal role in mediating SIRT1‐dependent EMT in trophoblasts.

## 3 DISCUSSION

Cumulative evidence has shown that among the maternal and perinatal outcomes of AMA pregnancies, there is a high rate of pregnancy complications (Abel et al., 2002; Andersen et al., 2000; du Fosse et al., 2020; Paulson et al., 2002). Moreover, a recent retrospective cohort study reported that maternal age over 40 years is an independent risk factor for adverse pregnancy outcomes (Marozio et al., 2019). However, few studies have explained why AMA pregnancies are more prone to pregnancy complications. Cellular senescence is characterized by irreversible growth arrest and altered function (Campisi & d'Adda di Fagagna, 2007) and can be divided into two types: replicative senescence, a permanent state of cell growth arrest that occurs after a limited number of cell divisions owing to telomere attrition, and premature senescence, a term used to describe a senescence‐like state that can occur from aberrant oncogene expression.

Due to its short life span, senescence of the placenta is not the same as that of other organs and is much faster. Previous reports have defined senescence as a normal phenomenon of placental development, and trophoblastic senescence may be essential for maintaining placental function (Higuchi et al., 2019; Velicky et al., 2018). However, premature or accelerated senescence and aging can occur as a result of placental stress that can lead to placental and clinical pathology. Unlike the signature of programmed developmental senescence in the embryo, senescence in the placenta shares features of senescence induced by DNA damage and exhibits coordinated activation of p53−p21 regulatory pathways (Chuprin et al., 2013).

The sirtuin protein family is widely known to be involved in longevity regulation (Wu et al., 2018). Therefore, we sought to study whether sirtuins are involved in placental senescence. By unbiased examination of all sirtuins, we found that SIRT1 was the sole sirtuin to show differential expression between AMA and normal placentas. The expression of SIRT1 is diminished with aging in mice; in contrast, increased expression of SIRT1 is sufficient to extend lifespan in yeast, Caenorhabditis elegans, and mice (Chen et al., 2020). Previous findings reported that oocytes become more exposed and vulnerable to oxidative stress as they age, accompanied by oocyte quality decrease (Sasaki et al., 2019), and the accumulation of DNA damage and oxidative stress in human tissue also result in a decrease in SIRT1 expression (Alves‐Fernandes & Jasiulionis, 2019). Therefore, disturbed redox homeostasis could be the underlying mechanism of the loss of SIRT1 in AMA trophoblasts. Of sirtuin family members, SIRT1 plays the most important role in regulating senescence. SIRT1 functions in regulating senescence in a complex process and its protein level has been shown to decrease in human fibroblasts during senescence (Abdelmohsen et al., 2007). There is evidence that an increased acetylation level of p53 correlates with replicative or oncogene‐induced premature senescence (Pearson et al., 2000). When activated, p53 inhibits cell proliferation via activation of its transcriptional target p21 (Campisi & d'Adda di Fagagna, 2007) and thereby irreversibly blocks cell cycle entry.

Although SIRT1 is a class III histone deacetylase, numerous studies have shown that it also binds to and deacetylates nonhistone proteins, such as P53, in multiple cell types (Lee & Gu, 2013). Consistently, acetylation of P53 is enhanced in the AMA placenta; moreover, downstream of the P53 pathway, P21 is accordantly upregulated. In the human placenta, the accumulation of senescent trophoblast cells from early pregnancy may have detrimental consequences and plays a key role in the pathogenesis of a number of adverse pregnancy outcomes, such as FGR, PE, spontaneous preterm birth, and intrauterine fetal death (Sultana et al., 2018).

Intriguingly, we found that these differences existed since the very beginning of pregnancy, suggesting that the loss of SIRT1 in the AMA placenta may be derived from intrinsic factors in the embryo. Therefore, cross breeding of young and old mice was carried out. Our data clearly showed that maternal age is a determinant for SIRT1 expression and P53−P21 axis activation in the placenta. Most likely, because the paternal genome in a zygote undergoes active DNA demethylation before the first mitosis, epigenetic modifications in sperm are largely erased by reprogramming after fertilization (Arney et al., 2002; Jenkins & Carrell, 2012). Although the biological significance and mechanisms of this paternal epigenome remodeling are still unclear, it might be why paternal age has little impact on basal senescence in the placentas of offspring.

Lappas and colleagues reported that SIRT1 is mainly localized in CTBs (Lappas et al., 2011), while in the present study, we provided evidence that SIRT1 is also expressed in EVTs. During the first trimester of pregnancy, invasive EVTs migrate into the maternal decidua and even the first third of the myometrium, where they remodel spiral arteries by replacing maternal endothelial cells (Tarrade et al., 2001). Defective invasion of EVTs causes pregnancy complications, such as PE and FGR (DaSilva‐Arnold et al., 2015). We speculated that loss of function of SIRT1 may affect EVT migration and invasion. As expected, we found that *SIRT1* knockdown‐induced senescence and inhibited the migration and invasion of HTR8/SVneo cells. Nevertheless, a previous study reported that SIRT1 negatively regulates the migration and invasion of Swan 71 cells, an immortalized first‐trimester trophoblast cell line (Lee et al., 2019). Swan 71 cells were derived from the telomerase‐mediated transformation of a 7‐week CTB isolate (Straszewski‐Chavez et al., 2009), and HTR8/SVneo cells were originally obtained from a human first‐trimester placenta and immortalized through transfection with a cDNA construct encoding the simian virus 40 large‐T antigen (Graham et al., 1993). Although both of these immortalized cell lines are widely used for the study of EVTs, neither of them perfectly shares all features of human primary trophoblasts. Thus, to further verify the findings from cell experiments, in this study, we generated trophoblast‐specific Sirt1‐deficient mouse placentas by mating transgenic mice harboring a Cre gene regulated by a trophoblast‐specific promoter with *Sirt1* ^fl/fl^ mice. The obtained *Elf5* ^cre/+^, *Sirt1* ^fl/fl^ placentas presented significantly higher levels of biomarkers of senescence and a diminished Jz area. Consequently, the weights of Sirt1‐deficient placentas and the body weights of the corresponding fetuses were significantly lower than those of the WT placentas. Consistent with our findings, Park et al. found that SIRT1 is widely expressed in both the Jz and the labyrinth zone of E17.5 placentas of C57BL/6 mice (Park et al., 2020), while Rajan et al. reported that SIRT1 is localized in the nuclei of trophoblasts in all layers of placentas of 129/Sv mice and that labyrinthine trophoblasts more uniformly expressed SIRT1 than did those in the junctional zone (Arul Nambi Rajan et al., 2018). Using global *Sirt1*‐KO mice, the authors also observed that the absence of SIRT1 led to growth restriction of the embryo and a smaller placenta that showed abnormalities in both the labyrinthine layer and Jz. These facts revealed that SIRT1 is essential for placental development. However, in our study, trophoblast‐specific *Sirt1*‐KO produced a different phenotype than that of the mouse AMA model, as *Sirt1*‐KO mice exhibited restricted growth of the fetus and placenta, whereas AMA mice retained the weight of individual fetuses and placental weight but showed resorption. A potential explanation for these findings is that the AMA mouse undergoes a natural aging process that subsequently results in systemic senescence in not only the placenta but also the egg, uterus, and other endocrine organs. However, with aging, endocrine signaling and metabolism will gradually change to adapt to and compensate for the aging of tissues and organs (Azzu & Valencak, 2017; van den Beld et al., 2018). On the other hand, although the generated trophoblast‐specific *Sirt1*‐KO mouse model displays premature senescence in the placenta, its global endocrine signaling and metabolism may not be able to compensate for such deterioration in the placenta due to the tissue‐specific SIRT1 deletion. Consequently, the phenotypes of these two models are not completely identical. Nevertheless, both models clearly present trophoblast senescence and dysfunction, as well as associated maldevelopment of the placenta.

To further explore the molecular basis underlying the regulatory role of SIRT1 in trophoblast mobility, the proteins binding to SIRT1 in both HTR8/SVneo cells and human first‐trimester villi were profiled by IP‐MS analyses, which revealed that vimentin could be a downstream effector of SIRT1 in trophoblasts. As a type III intermediate filament protein, vimentin contributes to subcellular and tissue‐specific biological functions and plays an important role in regulating EMT, which is widely thought to be an important mechanism contributing to the migration and metastasis of numerous cancer cells (Gao et al., 2018; Li et al., 2011). During placental development, trophoblasts undergo partial EMT, losing their organized epithelial phenotype and acquiring a migratory and invasive mesenchymal phenotype, which allows them to migrate to and infiltrate the maternal decidua and vessels (Davies et al., 2016; Imakawa et al., 2017). Most importantly, vimentin undergoes various functionally important PTMs, including acetylation (Kim et al., 2006), and one sirtuin family member, SIRT5, was found to physically interact with and deacetylate vimentin at K120 and thus influence the EMT process in hepatocellular carcinoma cells (Guo et al., 2018). Vimentin is a type III intermediate filament, one of the most conserved and abundant proteins in the intermediate filament protein family. Intermediate filament proteins are known to have ~1000–1700 acetylation targets based on the identification of their putative acetylation sites through large‐scale studies (Choudhary et al., 2009; Snider & Omary, 2014). Therefore, instead of looking at a specific acetylation site on vimentin, in this study, we evaluated the overall acetylation status of vimentin in response to SIRT1 expression or activation. Our results clearly showed that SIRT1 deacetylates vimentin in trophoblasts through protein−protein interactions and that EMT is impaired in the absence of SIRT1. Such inhibitory effects of SIRT1 deficiency on vimentin deacetylation and EMT were further validated in the *Sirt1*‐KO mouse placenta and first‐trimester villi of AMA pregnancies. Interestingly, we observed that the protein levels of SIRT1 and vimentin were correlated in trophoblasts, indicating that the acetylation of vimentin might influence its stability. Fatty acid synthase (FASN) acetylation enhances its association with the E3 ubiquitin ligase TRIM21 and promotes its degradation via the ubiquitin−proteasome pathway (Lin et al., 2016). Acetylation of the pyruvate kinase PKM targets it for degradation through chaperone‐mediated autophagy (Lv et al., 2011). It has long been believed that lysine acetylation can induce proteasome‐dependent and proteasome‐independent protein degradation, so the acetylation of vimentin may be the first step in activating its downstream protein degradation pathway (Narita et al., 2019; Sang et al., 2017). Therefore, SIRT1 may improve trophoblast EMT through deacetylation‐dependent stabilization of vimentin, although further investigation is warranted. To the best of our knowledge, the present study is the first to report SIRT1 regulation of trophoblast EMT through the modulation of vimentin acetylation.

In summary, AMA causes loss of SIRT1 in the placenta beginning at placentation, which results in premature senescence of trophoblasts and increased acetylation of vimentin, therefore compromising the invasion and migration of trophoblasts by suppressing EMT. Our data provide in‐depth insight into the critical role of SIRT1 in the regulation of trophoblast functions, as well as a potential interventional target for improving placental development and perinatal outcomes in AMA pregnancies.

## 4 MATERIALS AND METHODS

### 4.1 Ethics statement

The study design involving patients and animals was approved by the local ethical committee of the First Affiliated Hospital of Chongqing Medical University (No: 2017‐056) and is in accordance with the principles set out in the Declaration of Helsinki. All clinical samples were collected with written informed consent provided by the participating patients. The animals were regularly checked by qualified administrators for health monitoring, environmental supervision, animal welfare checks, and operational revision.

### 4.2 Patient recruitment and sample collection

Term placental samples were collected within 15 min of cesarean deliveries for nonmedical reasons, and none of the patients experienced labor prior to their c‐section. Twenty AMA (age ≥40 years) pregnancies and 25 control (age <30 years) pregnancies between 37 and 40 weeks of gestation were included in this study. First‐trimester villi samples were collected from subjects who legally and voluntarily terminated their pregnancies for nonmedical reasons between 6 and 8 weeks of gestation by using artificial abortion vacuum aspiration, including 25 AMA patients (age ≥40 years) and 30 controls (age <30 years). Patients with major complications, such as pre‐existing hypertension, GDM, premature rupture of membranes, renal diseases, premature labor, and PE, and in vitro fertilization and embryo transfer (IVF‐ET) pregnancies, were excluded. The patients’ clinical characteristics are shown in Tables S1 and S2. Placentas from the second trimester (14, 17, 21, and 24 weeks of gestational age, n = 3, respectively) were collected from women who underwent legal termination for nonmedical reasons. Each sample was divided into four portions: a portion was fixed in 4% paraformaldehyde and then embedded in paraffin; a portion was dehydrated and then transferred to OCT compound (Tissue‐Tek^®^ OCT Compound, SAKURA, USA); a portion was kept in RNAlater (Thermo Fisher Scientific, Waltham, USA); and the rest was immediately snap‐frozen in liquid nitrogen and stored at −80°C.

### 4.3 Mouse model of AMA

Female (n = 60) and male C57BL/6N mice aged 24 to 32 weeks old were purchased from the Charles River Lab (Vital River Laboratory Animal Technology, Inc. Beijing, China), while 8‐ to 12‐week‐old C57BL/6N female (n = 60) and male mice were assigned to the young group. The observation of a vaginal plug after the day of mating was considered E0.5. All mice were kept in a temperature‐controlled room (23°C) with a 12:12‐h light−dark cycle. Pregnant females were dissected at the indicated gestational age and processed for further experiments, including morphology recording, sample collection, and related measurements. Sample collection was in accordance with clinical samples.

### 4.4 Establishment of trophoblast‐specific Sirt1‐deficient mice

*Elf5* ^cre/+^ mice were generous gifts from Prof. Haibin Wang, Xiamen University, China (Kong et al., 2018). *Sirt*1^fl/fl^ mice [B6.129‐*Sirt*1^tm3Fwa^/DsinJ] were purchased from The Jackson Laboratory. Genotyping PCR was performed using genomic DNA extracts from mouse tail biopsies. *Sirt1* ^fl/fl^ mice were bred with *Elf5* ^cre/+^ mice to generate *Elf5* ^cre/+^, *Sirt1* ^fl/fl^ males. These mice were then bred with *Sirt1* ^fl/fl^ females to generate trophoblast‐specific *Sirt1*‐KO placentas.

### 4.5 Cell culture

The immortalized human trophoblast cell line HTR8/SVneo was purchased from American Type Culture Collection (ATCC, Inc., Manassas, USA) and cultured in Roswell Park Memorial Institute (RPMI) 1640 serum‐free cell freezing medium with L‐glutamine (RPMI 1640, #11875093, Gibco, Carlsbad, USA) supplemented with 10% FBS (#ST30‐2602, Adenbach, Germany). All cells were incubated under standard culturing conditions (37°C and 5% CO_2_ in a humidified atmosphere). SRT1720 (#HY‐10532, MedChemExpress, USA) was dissolved in dimethyl sulfoxide (DMSO) (Sigma), and NAM (#HY‐B0150, MedChemExpress, USA) was dissolved in double‐distilled water.

### 4.6 Lentivirus infection

Lentiviruses carrying short hairpin RNA (shRNA) targeting human sirtuin1 (*SIRT1*) lentivirus vectors (GV248) were purchased from GeneChem, Inc. (Shanghai, China). A total of 4 × 10^5^ HTR8/SVneo cells were transfected with 2 × 10^6^ viruses (multiplicity of infection = 20) for 48 h in the presence of HiTransG A infection enhancer (GeneChem, Shanghai, China) according to the manufacturer's instructions. Then, the cells were transferred to fresh medium containing puromycin (1 μg/ml) for the selection of stable clones after three passages. The shRNA sequence was 5’‐GGCTTGATGGTAATCAGTA‐3’.

### 4.7 siRNA‐mediated silencing

The scrambled control siRNA (NC‐siRNA) (5’‐UUCUCCGAACGUGUCACGUTT‐3’) and siRNA‐vimentin (5’‐GCAUCACGAUGACCUUGAAUA‐3’) were synthesized by GenePharma Inc. (Shanghai, China) and transfected into HTR8/SV neo cells using EndoFectin^TM^ Max (CA#EF013, GeneCopoeia Inc., Rockville, USA) according to the manufacturer's instructions. All cells were cultured for 48 h after transfection before receiving other treatments. Interference efficiency was verified using western blotting.

### 4.8 Immunohistochemistry

The placental and decidual tissues were washed with PBS and fixed overnight with 4% paraformaldehyde at room temperature. Then, the samples were dehydrated and embedded in paraffin before sectioning into 4‐μm‐thick sections. For IHC, the sections were deparaffinized in xylene, rehydrated in a serial ethanol gradient (100%–95%–85%–75%), and then heated in a microwave in sodium citrate (10 mM, pH 6.0) for 15 min to retrieve antigens. Sections were blocked with 3% peroxide in methanol at room temperature for 10 min to inhibit the activity of endogenous peroxidases. Afterward, sections were incubated with primary antibodies against SIRT1 (1:100; Proteintech, #13161, Wuhan, China), CK7 (1:100; #ab68459, Abcam, Cambridge, UK), HLA‐G (1:100; Proteintech, #66447, Wuhan, China), or Tpbpa (1:100; Bioss, #17153R, Beijing, China) at 4°C overnight. Then, a secondary antibody conjugated with horseradish peroxidase was applied and incubated for 1 h at room temperature, followed by development with a diaminobenzidine solution. The images were captured with an Evos Fl Color Imaging System (Thermo Fisher Scientific, USA).

### 4.9 Immunofluorescence

Immunostaining was performed on frozen sections of human decidua, human term placentas, and mouse placentas as described previously (Yuan et al., 2018). Briefly, frozen placental sections and trophoblast cells were permeabilized with 0.2% Triton X‐100 (#ST797, Beyotime, Beijing, China) and blocked with 1% bovine serum albumin (BSA) (#ST023, Beyotime, Beijing, China). The tissues and cells were then incubated with primary antibodies against SIRT1 (#8469, Cell Signaling Technology, Boston, USA) and CK7 (#ab68459, Abcam, Cambridge, UK)/HLA‐G (Proteintech, #66447, Wuhan, China) at 4°C overnight, followed by incubation with a FITC‐conjugated or cyanine 3‐conjugated goat anti‐rabbit secondary antibody (Proteintech, #SA0009‐2, Wuhan, China) for 1 h at room temperature. The nuclei were stained with 4’,6‐diamidino‐2‐phenylindole (DAPI), and images were visualized using an Evos Fl Color Imaging System (Thermo Fisher Scientific, Waltham, USA).

### 4.10 Western blotting

Protein extracts were prepared from placental tissues and cells using RIPA lysis buffer supplemented with 1% cocktail phosphatase inhibitor and 1% cocktail protease inhibitor (Bimake, Houston, USA). The lysates were then separated by SDS‐PAGE (Bio‐Rad) and transferred onto PVDF membranes (Millipore Sigma). After blocking with 5% nonfat dry milk (Bio‐Rad) in Tris buffer containing 5% Tween‐20 for 1 h at room temperature, the membranes were immunoblotted with primary antibodies against SIRT1 (#8469, Cell Signaling Technology, Boston, USA), SIRT2~7, P53 (#10442‐1, Proteintech, Wuhan, China), P21 (#SC817, Santa Cruz Biotechnology, USA), acetylated P53 (#2525, Cell Signaling Technology, Boston, USA), vimentin (#10366‐1, Proteintech, Wuhan, China), N‐cadherin (#66219‐1, Proteintech, Wuhan, China), E‐cadherin (#20874‐1, Proteintech, Wuhan, China), SNAIL (#13099‐1, Proteintech, Wuhan, China), or β‐actin (#66009‐1, Proteintech, Wuhan, China) overnight at 4°C. After rinsing with Tris buffer containing 5% Tween‐20, the membranes were then incubated with the corresponding horseradish peroxidase‐conjugated secondary antibodies (ZSGB‐BIO) at room temperature for 1 h. Immunoreactive bands were developed using an enhanced chemiluminescent substrate (Millipore Sigma, USA), and the images were captured and analyzed by a ChemiDoc XRS+system (Bio‐Rad, USA).

### 4.11 Matrigel invasion assay

The invasion assay was performed in a transwell chamber consisting of a 24‐well plate with membrane inserts (Corning, New York, USA) containing 8‐mm‐pore‐sized polycarbonate filters precoated with 60 μl of 1 mg/ml Matrigel matrix solution (BD Biosciences, San Jose, USA). A suspension of 4 × 10^4^ cells in 200 μl of serum‐free culture medium was added to the inserts, and each insert was placed in the lower chamber containing 600 μl of 10% fetal bovine serum (FBS) culture medium. After 24 h, the cells that penetrated the membrane were fixed with 4% paraformaldehyde and stained with crystal violet. Images were captured by using the Evos Fl Color Imaging System (Thermo Fisher Scientific, Waltham, USA).

### 4.12 Wound‐healing assay

HTR8/SVneo cells were seeded onto 6‐well plates and grown to more than 90% confluence, and then a scratch on the cell monolayer was made using a pipette tip. The cells were then rinsed twice with fresh culture medium and allowed to stay in culture for another 24 h, and pictures were taken at 0, 12, and 24 h. The area of wound healing was measured with ImageJ 1.50i software (https://imagej.en.softonic.com/). Each experiment was performed in triplicate.

### 4.13 RNA extraction and RT‐qPCR

Total RNA was extracted from the cultured cells, villous tissues, and placental tissues using TRIzol reagent (Invitrogen, Carlsbad, USA) according to the manufacturer's instructions. The RNA concentration was measured by ultraviolet spectroscopy (Nanodrop 2000; Thermo). Total RNA (1 μg) was used for reverse transcription with a Prime Script RT reagent kit (Roche Life Science, Mannheim, Germany). Primers were synthesized by TSINGKE Biological Technology; GAPDH was used as an endogenous control for gene expression analysis. The sequences of the PCR primer pairs for each gene are shown in Table S3. Quantitative RT‐PCR was carried out using a Bio‐Rad CFX Connect Detection System (Bio‐Rad, Hercules, USA). PCR cycling conditions included predenaturing at 95°C for 3 min, followed by 40 cycles (94°C for 5 s, 58°C for 15 s, and 72°C for 15 s) and extension at 72°C for 30 s. The threshold cycle Ct value was defined as the fractional cycle number at which the fluorescence passed the fixed threshold.

### 4.14 NAD^+^/NADH assay

NAD^+^/NADH levels were measured with Amplite Fluorimetric NAD/NADH ratio assay kits (ATT Bioquest, CA) as previously described (Li et al., 2015). Briefly, ~20 mg human placental or mouse placental tissue was weighed, washed with cold PBS, and homogenized with 400 μl of lysis buffer in a microcentrifuge tube. After centrifugation at 1,000 *g* for 5−10 min, the supernatant was collected and used for subsequent NAD^+^/NADH assays. For the measurement of intracellular NAD^+^/NADH levels, 25 μl of cell lysate was treated with or without NAD^+^/NADH extraction solution for 15 min, neutralized with extraction solutions at room temperature, and incubated at room temperature in the dark for 30 min after the addition of 75 μl of NADH reaction mixture. The readings were taken by running a 96‐well black plate on a Varioskan LUX fluorescence microplate reader (Thermo Fisher Scientific, Waltham, USA) at Ex/Em = 530–570/590–600 nm (maximum Ex/Em = 540/590 nm). The blank signal was subtracted from the values for those wells with the NADH reactions.

### 4.15 Senescence‐associated β‐galactosidase staining

Senescence‐associated β‐galactosidase staining was performed on frozen sections of tissues and confluent HTR8/SVneo cells in 6‐well plates using a Senescence β‐Galactosidase Staining Kit (#C0602, Beyotime Biotechnology, Beijing, China) according to the manufacturer's instructions. The presence of β‐galactosidase at pH 6.0 is a known characteristic of senescent cells and is absent from presenescent, proliferative, quiescent, or immortal cells (Dimri et al., 1995). Briefly, frozen tissue sections and trophoblast cells were fixed in fixative solution for 15 min at room temperature and stained overnight at 37°C in air in a nonhumidified incubator. Cell plates and tissue sections were sealed to prevent evaporation. After 24 h of incubation, the cells and tissue sections were observed using phase‐contrast microscopy. The images were captured by an Evos Fl Color Imaging System (Thermo Fisher Scientific, Waltham, USA).

### 4.16 Villous explant culture

The placental villous tissues were dissected into explants of 2–5 mm in diameter and explanted as previously described (Chen et al., 2018; Zhang et al., 2012). A 24‐well plate was precoated with 50 μl of a 1 mg/ml Matrigel matrix solution (CA# 354234, BD Biosciences, San Jose, CA, USA) and incubated at 37°C for 4 h for solidification. Serum‐free DMEM/F12 (Gibco) medium containing 10% FBS (PAN) with 20 mM NAM (#HY‐B0150, MedChemExpress, USA), 3 μM SRT1720 (#HY‐10532, MedChemExpress, USA), 500 nmol/l lentiviral vector‐based shRNA targeting SIRT1 or an equal concentration of scrambled shRNA (GeneChem, Shanghai, China) was added to the wells; the explants were cultured in 3% oxygen and 5% CO_2_ for 48 h. The explants with good attachment and outgrowth on the gel were assessed after 24 and 48 h, respectively. The evaluation of villus outgrowth was performed as previously described (Li et al., 2014).

### 4.17 Coimmunoprecipitation

Anti‐SIRT1 antibody (Cell Signaling Technology, #8469), anti‐vimentin antibody (Proteintech, #10366‐1), or matched IgG isotype antibody (Cell Signaling Technology, #5946) were incubated with Protein A/G Magnetic Beads (Bimake, Houston, USA, #B23202) for 1.5 h at room temperature. Cells and tissues were lysed with IP lysis buffer (Thermo Fisher Scientific, Waltham, USA, #87787), and a total of 400 μg of each sample lysate was incubated with an antibody−bead complex for another 8 h. Then, the protein extracts were precipitated by the antibody−bead complex using a magnetic rack. After being washed 3 times with IP lysis buffer, Co‐IP products were boiled at 95°C for 15 min with diluted 4 × Laemmli protein sample buffer (Bio‐Rad, Hercules, USA, #1610747) in lysis buffer. Finally, proteins were resolved by SDS/PAGE and immunoblotted with antibodies as indicated.

### 4.18 LC‐MS/MS

HTR8/SVneo cells and human early pregnancy villi were lysed with IP lysis buffer (Thermo Fisher Scientific, Waltham, USA, #87787). Soluble fractions were subjected to IP. Anti‐SIRT1 antibody (Cell Signaling Technology, #8469) and a matched IgG isotype antibody (Cell Signaling Technology, #5946) were incubated with Protein A/G Magnetic Beads (Bimake, Houston, USA, #B23202) for 1.5 h at room temperature, and then, the cell and tissue lysates were incubated with an antibody−bead complex for another 8 h at 4°C. The protein complex‐containing beads were washed four times with PBS, and then, proteins were eluted, boiled in loading buffer (containing 1% SDS), and resolved by SDS/PAGE. Coomassie blue staining was used to visualize the protein bands, and the protein bands in the whole lane of the gel were then excised for in‐gel tryptic digestion. The peptides were subjected to an NSI source followed by tandem mass spectrometry (MS/MS) in Q Exactive™ Plus (Thermo Fisher Scientific, Waltham, USA) coupled online to an EASY‐nLC 1000 UPLC system (Thermo Fisher Scientific, Waltham, USA). The details are provided in the supplementary methods.

### 4.19 Statistical analysis

Data are presented as the mean ± SEM. Statistical data were analyzed by Student's t‐test (2 groups) and ANOVA (>2 groups). The statistical analyses were performed by using GraphPad Prism software (version 7.0; La Jolla, California, USA). A *p*‐value <0.05 was considered significant.

## CONFLICT OF INTEREST

The authors have no conflicts of interest to declare.

## AUTHORS’ CONTRIBUTIONS

CT conceived and designed the study; LX, XY, ZC, HF, SL, PX, JY, and LW performed the experiments and analyzed the data; RG, YF, RS, MK, and PB interpreted the results; CT and HQ obtained funding; LX and CT wrote the draft; HQ, MK, and PB edited the manuscript.

## Supporting information

Supplementary Material

## Data Availability

The data and materials described in the manuscript will be available upon reasonable request to the corresponding authors; delivery charges and a material transfer agreement may apply. The proteomics data reported in this paper have been deposited in a public data depository under accession number PXD026574 and are publicly accessible at https://www.iprox.org.
