# Supplementary material for: Advanced Maternal Age‐associated SIRT1 Deficiency Compromises Trophoblast Epithelial−Mesenchymal Transition through an Increase in Vimentin Acetylation
Source: Aging Cell. 2021 Oct 3;20(10):e13491. doi: 10.1111/acel.13491 (PMC8520724; doi:10.1111/acel.13491)
Supplement: Supplementary file 1 — Supplementary Material [file ACEL-20-e13491-s001.docx]

**Supplementary Data**

**Table S1.** Clinical characteristics of the patients who provided term placental tissue.

| Parameters | Control  (n=25) | Advanced maternal age  (n=20) |
| --- | --- | --- |
| Age (years) | 27.24±0.38 | 41.15±0.32**** |
| Gestational age at delivery (weeks) | 39.07±0.12 | 38.79±0.23 |
| Body mass index  (BMI; kg/m^2^) | 25.98±0.51 | 26.42±0.56 |
| Gravidity | 2.960±0.38 | 3.300±0.34 |
| Parity (%)  0  1  ≥2 | 11 (44%)  14 (56%)  0 (0%) | 6 (30%)  13 (65%)  1 (5%) |
| Systolic blood pressure  (mmHg) | 114.4±2.37 | 109.3±2.05 |
| Diastolic blood pressure  (mmHg) | 71.83±2.12 | 67.00±1.53 |

Data are expressed as the mean ± SEM. The data were analyzed by Student’s t-test. ****P＜0.0001.

**Table S2.** Clinical characteristics of the patients who provided villi extracts.

| **Parameters** | **Control**  **(n=30)** | **Advanced Maternal Age**  **(n=25)** |
| --- | --- | --- |
| **Age** (years) | 24.67±0.50 | 41.84±0.40**** |
| **Gestational age at delivery** (weeks) | 7.083±0.14 | 6.976±0.14 |
| **Body mass index**  (BMI; kg/m^2^) | 21.21±0.35 | 21.71±0.34 |
| **Gravidity** | 2.000±0.17 | 2.440±0.18 |
| **Parity** (%)  0  1  ≥2 | 17 (57%)  10 (33%)  3 (10%) | 5 (20%)  15 (60%)  5 (20%) |
| **Systolic blood pressure**  (mmHg) | 114.5±1.16 | 117.5±1.53 |
| **Diastolic blood pressure**  (mmHg) | 75.80±1.42 | 77.32±1.29 |

Data are expressed as the mean ± SEM. The data were analyzed by Student’s t-test. ****P＜0.0001.

**Table S3.** Primers used for RT-qPCR amplification.

| **Primers** | **Sequences (5’-3’)** |
| --- | --- |
| *SIRT1* | Forward GGAACCTTTGCCTCATCTACA  Reverse CACTAGCCTATGACACAACTC |
| *P53* | Forward AGGGATGTTTGGGAGATGTAAG  Reverse CCTGGTTAGTACGGTGAAGTG |
| *P21* | Forward CGGAACAAGGAGTCAGACATT  Reverse AGTGCCAGGAAAGACAACTAC |

**Table S4.** Primers used for PCR amplification and PCR genotyping of the *Sirt1*^loxp/loxp^ and *Elf5*-Cre transgenes.

| **Primers** | **Sequences (5’-3’)** |
| --- | --- |
| *Elf5*-F  *Elf5*-R | GAATTCTGGAGCGGGTTGAC  TAATGGTGCAACGGGTCCTC |
| *CRE*-98R  *Sirt1*-Common  *Sirt1*-Wild type | TTCTTGCGAACCTCATCACTC  AGGAATCCCACAAGGAGACAG  GAATTCTGGAGCGGGTTGAC |
|  |  |

**Table S5.** Identification of SIRT1-binding proteins in HTR8/SVneo cells by LC-MS/MS.

| **Proteins identified in HTR8/SVneo cells** | **Gene names** | **MW [kDa]** | **Protein score** |
| --- | --- | --- | --- |
| RNA-binding motif protein, X chromosome | RBMX | 42.31 | 386.49 |
| Vimentin | VIM | 53.62 | 336.96 |
| Heterogeneous nuclear ribonucleoprotein R | HNRNPR | 70.90 | 319.00 |
| RNA-splicing ligase RtcB homolog | RTCB | 55.17 | 315.26 |
| Unconventional myosin-Ic | MYO1C | 121.61 | 278.05 |
| Heterogeneous nuclear ribonucleoprotein Q | SYNCRIP | 69.56 | 264.26 |
| Heat shock cognate 71 kDa protein | HSPA8 | 70.85 | 253.41 |
| Tubulin beta-4B chain | TUBB4B | 49.80 | 250.38 |
| Heterogeneous nuclear ribonucleoprotein U | HNRNPU | 90.53 | 247.46 |
| Tubulin beta chain | TUBB | 49.64 | 236.88 |
| Myosin-14 | MYH14 | 227.73 | 233.04 |
| RNA-binding protein EWS | EWSR1 | 68.44 | 205.43 |
| Nucleolin | NCL | 76.57 | 202.83 |
| Heat shock protein HSP 90-beta | HSP90AB1 | 83.21 | 202.47 |
| KH domain-containing, RNA-binding, signal transduction-associated protein 1 | KHDRBS1 | 48.20 | 197.47 |
| ATP-dependent RNA helicase DDX1 | DDX1 | 82.38 | 197.04 |
| Serum albumin | ALB | 69.32 | 188.48 |
| Myosin regulatory light chain 12A | MYL12A | 19.78 | 187.78 |
| Protein FAM98A | FAM98A | 55.24 | 177.96 |
| Tubulin alpha-1C chain | TUBA1C | 49.86 | 176.99 |
| Keratin, type I cytoskeletal 14 | KRT14 | 51.53 | 169.10 |
| Splicing factor, proline- and glutamine-rich | SFPQ | 76.10 | 144.89 |
| Glyceraldehyde-3-phosphate dehydrogenase | GAPDH | 36.03 | 143.95 |
| RNA-binding protein FUS | FUS | 53.39 | 143.65 |
| Myosin light polypeptide 6 | MYL6 | 16.92 | 139.66 |
| TATA-binding protein-associated factor 2N | TAF15 | 61.79 | 137.05 |
| Tropomyosin beta chain | TPM2 | 32.83 | 132.40 |
| Elongation factor 1-alpha 1 | EEF1A1 | 50.11 | 127.76 |
| Tropomyosin alpha-4 chain | TPM4 | 28.50 | 110.93 |
| E3 ubiquitin-protein ligase TRIM21 | TRIM21 | 54.14 | 110.40 |
| Pyruvate kinase PKM | PKM | 57.90 | 108.53 |
| Heterogeneous nuclear ribonucleoprotein H | HNRNPH1 | 49.20 | 108.02 |
| Non-POU domain-containing octamer-binding protein | NONO | 54.20 | 103.87 |
| Keratin, type II cytoskeletal 5 | KRT5 | 62.34 | 99.66 |
| Probable ATP-dependent RNA helicase DDX5 | DDX5 | 69.10 | 95.87 |
| Heterogeneous nuclear ribonucleoprotein U-like protein 2 | HNRNPUL2 | 85.05 | 93.95 |
| Lactotransferrin | LTF | 78.13 | 88.88 |
| Hemoglobin subunit alpha | HBA1 | 15.25 | 88.46 |
| rRNA 2'-O-methyltransferase fibrillarin | FBL | 33.76 | 84.87 |
| Eukaryotic initiation factor 4A-I | EIF4A1 | 46.12 | 76.05 |
| Protein S100-A9 | S100A9 | 13.23 | 73.99 |
| LIM domain and actin-binding protein 1 | LIMA1 | 85.17 | 73.83 |
| Heterogeneous nuclear ribonucleoprotein K | HNRNPK | 50.94 | 72.88 |
| Coronin-1C | CORO1C | 53.22 | 69.37 |
| Unconventional myosin-XVIIIa | MYO18A | 232.97 | 67.83 |
| Neprilysin | MME | 85.46 | 67.29 |
| CD44 antigen | CD44 | 81.49 | 64.99 |
| Dermcidin | DCD | 11.28 | 63.15 |
| ATP-dependent RNA helicase DDX39A | DDX39A | 49.10 | 60.01 |
| Cofilin-1 | CFL1 | 18.49 | 58.94 |
| Splicing factor U2AF 65 kDa subunit | U2AF2 | 53.47 | 56.27 |
| Thioredoxin | TXN | 11.73 | 55.91 |
| ATP-dependent RNA helicase DDX3Y | DDX3Y | 73.11 | 55.59 |
| Elongation factor 1-gamma | EEF1G | 50.09 | 54.51 |
| ATP synthase subunit alpha, mitochondrial | ATP5F1A | 59.71 | 53.13 |
| Prohibitin | PHB | 29.79 | 53.08 |
| Hemoglobin subunit delta | HBD | 16.05 | 52.71 |
| Heterogeneous nuclear ribonucleoprotein D0 | HNRNPD | 38.41 | 51.63 |
| Polyubiquitin-C | UBC | 76.99 | 50.86 |
| Ceruloplasmin | CP | 122.13 | 50.42 |
| Histone H4 | H4C1 | 11.36 | 50.37 |
| Protein S100-A8 | S100A8 | 10.83 | 48.86 |
| 60S ribosomal protein L13 | RPL13 | 24.25 | 48.17 |
| Immunoglobulin lambda-like polypeptide 5 | IGLL5 | 23.05 | 47.31 |
| Lysozyme C | LYZ | 16.53 | 46.82 |
| 60 kDa heat shock protein, mitochondrial | HSPD1 | 61.02 | 46.29 |
| Hydroxyacid oxidase 2 | HAO2 | 38.81 | 46.27 |
| Protein SET | SET | 33.47 | 45.76 |
| Bcl-2-associated transcription factor 1 | BCLAF1 | 106.06 | 44.60 |
| Zinc-finger and BTB domain-containing protein 49 | ZBTB49 | 85.01 | 44.60 |
| U6 snRNA-associated Sm-like protein LSm4 | LSM4 | 15.34 | 39.98 |

**Table S6.** Identification of SIRT1-binding proteins in human villi by LC-MS/MS.

| **Proteins identified in human villi** | **Gene names** | **MW [kDa]** | **Protein score** |
| --- | --- | --- | --- |
| Alpha-actinin-4 | ACTN4 | 104.79 | 588.44 |
| Tropomyosin alpha-4 chain | TPM4 | 28.50 | 542.36 |
| Gelsolin | GSN | 85.64 | 497.42 |
| Actin, aortic smooth muscle | ACTA2 | 41.98 | 452.48 |
| Vimentin | VIM | 53.62 | 403.34 |
| Plectin | PLEC | 531.47 | 287.23 |
| Alpha-actinin-1 | ACTN1 | 102.99 | 240.44 |
| TATA-binding protein-associated factor 2N | TAF15 | 61.79 | 238.58 |
| RNA-binding protein EWS | EWSR1 | 68.44 | 234.03 |
| E3 ubiquitin-protein ligase TRIM21 | TRIM21 | 54.14 | 207.29 |
| Immunoglobulin heavy constant gamma 4 | IGHG4 | 35.92 | 199.79 |
| Protein-glutamine gamma-glutamyltransferase 2 | TGM2 | 77.28 | 172.40 |
| Tubulin alpha-1C chain | TUBA1C | 49.86 | 170.14 |
| Immunoglobulin kappa variable 3-20 | IGKV3-20 | 12.55 | 166.77 |
| Cholesterol side-chain cleavage enzyme, mitochondrial | CYP11A1 | 60.06 | 165.20 |
| Keratin, type II cytoskeletal 5 | KRT5 | 62.34 | 160.39 |
| Immunoglobulin lambda variable 3-9 | IGLV3-9 | 12.32 | 158.02 |
| Immunoglobulin heavy constant gamma 3 | IGHG3 | 41.26 | 156.62 |
| Immunoglobulin kappa variable 2D-29 | IGKV2D-29 | 13.13 | 154.23 |
| Immunoglobulin lambda constant 2 | IGLC2 | 11.29 | 153.86 |
| Myosin light polypeptide 6 | MYL6 | 16.92 | 153.34 |
| Immunoglobulin kappa variable 2D-28 | IGKV2D-28 | 12.95 | 152.51 |
| Immunoglobulin kappa variable 2-30 | IGKV2-30 | 13.18 | 146.06 |
| Immunoglobulin lambda-1 light chain | Immunoglobulin | 22.82 | 141.11 |
| Endoplasmic reticulum chaperone BiP | HSPA5 | 72.29 | 140.98 |
| Heterogeneous nuclear ribonucleoprotein U | HNRNPU | 90.53 | 134.03 |
| Hemoglobin subunit alpha | HBA1 | 15.25 | 131.56 |
| Splicing factor, proline- and glutamine-rich | SFPQ | 76.10 | 130.74 |
| Adseverin | SCIN | 80.44 | 127.89 |
| Tropomyosin alpha-3 chain | TPM3 | 32.93 | 125.38 |
| Elongation factor 1-alpha 1 | EEF1A1 | 50.11 | 124.56 |
| Immunoglobulin heavy constant mu | IGHM | 49.41 | 121.85 |
| Immunoglobulin lambda constant 7 | IGLC7 | 11.25 | 119.16 |
| Immunoglobulin kappa variable 1-6 | IGKV1-6 | 12.69 | 117.88 |
| Tubulin beta chain | TUBB | 49.64 | 117.40 |
| Major vault protein | MVP | 99.27 | 101.54 |
| Heterogeneous nuclear ribonucleoprotein R | HNRNPR | 70.90 | 98.07 |
| Immunoglobulin heavy variable 3-33 | IGHV3-33 | 13.07 | 95.81 |
| Immunoglobulin kappa variable 1-17 | IGKV1-17 | 12.77 | 95.65 |
| Myosin regulatory light chain 12A | MYL12A | 19.78 | 90.96 |
| Immunoglobulin kappa variable 1-33 | IGKV1-33 | 12.84 | 89.28 |
| Heterogeneous nuclear ribonucleoprotein H | HNRNPH1 | 49.20 | 87.06 |
| Immunoglobulin kappa variable 6D-21 | IGKV6D-21 | 12.33 | 85.51 |
| Immunoglobulin heavy variable 5-10-1 | IGHV5-10-1 | 12.76 | 82.98 |
| Coronin-1C | CORO1C | 53.22 | 78.88 |
| Heat shock protein HSP 90-beta | HSP90AB1 | 83.21 | 78.46 |
| Reticulocalbin-1 | RCN1 | 38.87 | 77.84 |
| Immunoglobulin heavy variable 4-28 | IGHV4-28 | 13.12 | 75.73 |
| Ceruloplasmin | CP | 122.13 | 75.01 |
| 60S ribosomal protein L27a | RPL27A | 16.55 | 74.35 |
| Probable nonfunctional immunoglobulin kappa variable 3-7 | IGKV3-7 | 12.78 | 72.28 |
| Unconventional myosin-Id | MYO1D | 116.13 | 72.25 |
| Immunoglobulin lambda variable 1-47 | IGLV1-47 | 12.28 | 67.95 |
| Non-POU domain-containing octamer-binding protein | NONO | 54.20 | 66.35 |
| Immunoglobulin kappa variable 3D-11 | IGKV3D-11 | 12.62 | 66.14 |
| Immunoglobulin heavy variable 4-59 | IGHV4-59 | 12.93 | 65.83 |
| Immunoglobulin kappa variable 3D-15 | IGKV3D-15 | 12.53 | 65.41 |
| Immunoglobulin lambda variable 3-19 | IGLV3-19 | 12.03 | 65.20 |
| 3 beta-hydroxysteroid dehydrogenase/Delta 5-->4-isomerase type 1 | HSD3B1 | 42.23 | 64.98 |
| Immunoglobulin delta heavy chain | Immunoglobulin | 56.19 | 64.18 |
| Heterogeneous nuclear ribonucleoprotein Q | SYNCRIP | 69.56 | 62.46 |
| Immunoglobulin kappa variable 4-1 | IGKV4-1 | 13.37 | 61.49 |
| Immunoglobulin lambda variable 8-61 | IGLV8-61 | 12.81 | 58.70 |
| Histone H3.3C | H3-5 | 15.20 | 56.14 |
| Drebrin | DBN1 | 71.39 | 55.84 |
| Putative RNA-binding protein Luc7-like 2 | LUC7L2 | 46.49 | 55.03 |
| Solute carrier family 2, facilitated glucose transporter member 1 | SLC2A1 | 54.05 | 49.78 |
| Hemoglobin subunit delta | HBD | 16.05 | 49.38 |
| Dermcidin | DCD | 11.28 | 47.75 |
| Immunoglobulin heavy variable 1-69 | IGHV1-69 | 12.65 | 47.35 |
| Serpin A12 | SERPINA12 | 47.15 | 47.25 |
| Antithrombin-III | SERPINC1 | 52.57 | 46.68 |
| Putative uncharacterized protein PRO1854 | PRO1854 | 8.31 | 44.32 |
| Zinc-finger and BTB domain-containing protein 49 | ZBTB49 | 85.01 | 44.16 |
| Protein SHQ1 homolog | SHQ1 | 65.08 | 44.03 |
| Immunoglobulin heavy variable 3-49 | IGHV3-49 | 13.05 | 43.65 |
| Heat shock protein beta-1 | HSPB1 | 22.77 | 42.37 |
| 40S ribosomal protein S9 | RPS9 | 22.58 | 42.31 |
| Elongation factor-like GTPase 1 | EFL1 | 125.35 | 41.90 |
| Immunoglobulin heavy variable 3-15 | IGHV3-15 | 12.92 | 41.01 |
| Apoptotic chromatin condensation inducer in the nucleus | ACIN1 | 151.77 | 38.66 |
| Immunoglobulin heavy variable 1-46 | IGHV1-46 | 12.92 | 37.36 |
| Phospholipid-transporting ATPase ABCA1 | ABCA1 | 254.14 | 35.13 |
| Pregnancy-specific beta-1-glycoprotein 5 | PSG5 | 37.69 | 35.03 |
| Immunoglobulin heavy variable 2-5 | IGHV2-5 | 13.22 | 30.16 |

**
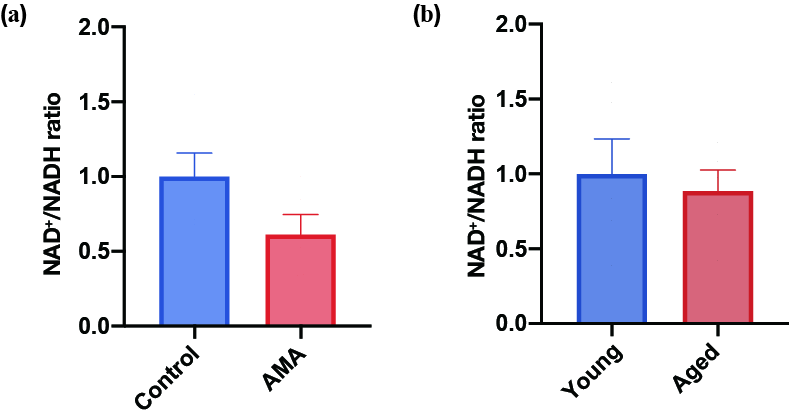
**

**Fig S1.** NAD^+^/NADH ratio in human and mouse placentas.

(a) NAD^+^/NADH ratio in human term placentas of the advanced maternal age (AMA) and young (control) groups; n=5. (b) NAD^+^/NADH ratio in placentas from young and aged mice; n=5. Data were analyzed using Student’s t-test. All data are presented as the mean ± SEM.

**
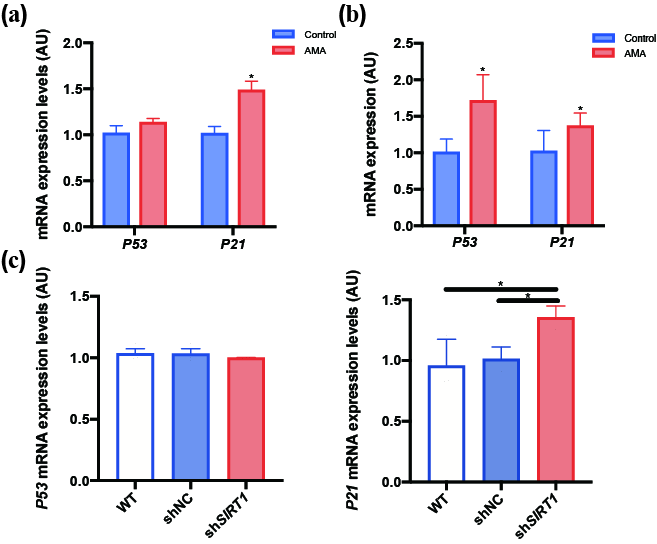
**

**Fig S2.** mRNA expression levels of *P53* and *P21* in human villi, term placenta and HTR8/SVneo cells.

(a) RT-qPCR assessment of *P53* and *P21* levels in human term placentas from the advanced maternal age (AMA) and young (Control) groups; n=3. (b) RT-qPCR assessment of *P53* and *P21* levels in human villi; n=3. (c) RT-qPCR assessment of *P53* and *P21* levels in shNC- or sh*SIRT1*-transfected HTR8/SVneo cells as well as wild-type (WT) cells. Data were analyzed using Student’s t-test (a, b) and one-way ANOVA (c), *P <0.05, **P <0.01. All data are presented as the mean ± SEM.


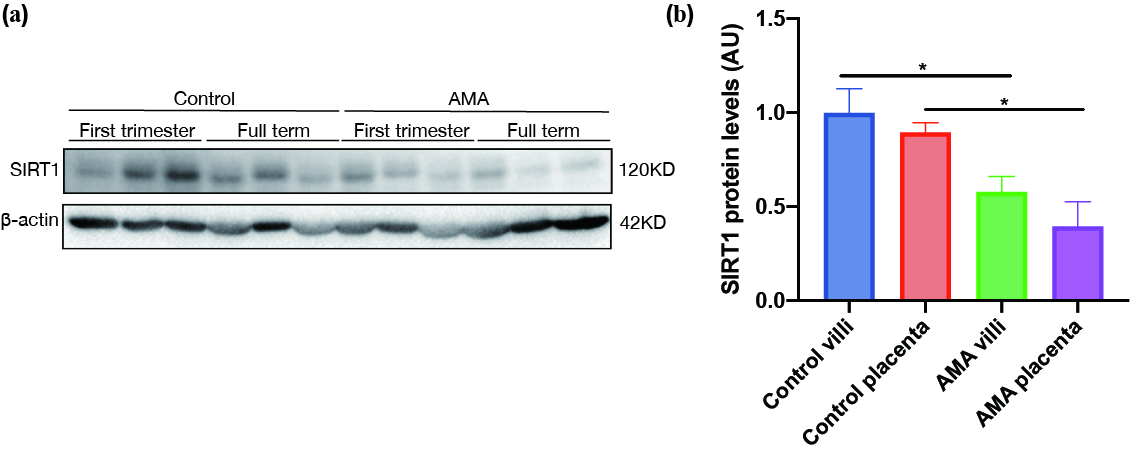


**Fig S3.** SIRT1 expression patterns in human placenta.

(a) Representative Western blots of SIRT1 in first-trimester villi and full-term placentas from control and AMA pregnancies; n=3. All experiments were performed in triplicate. (b) Statistical analysis of SIRT1 protein levels. Data were analyzed using Student’s t-test; *P <0.05. All data are presented as the mean ± SEM.


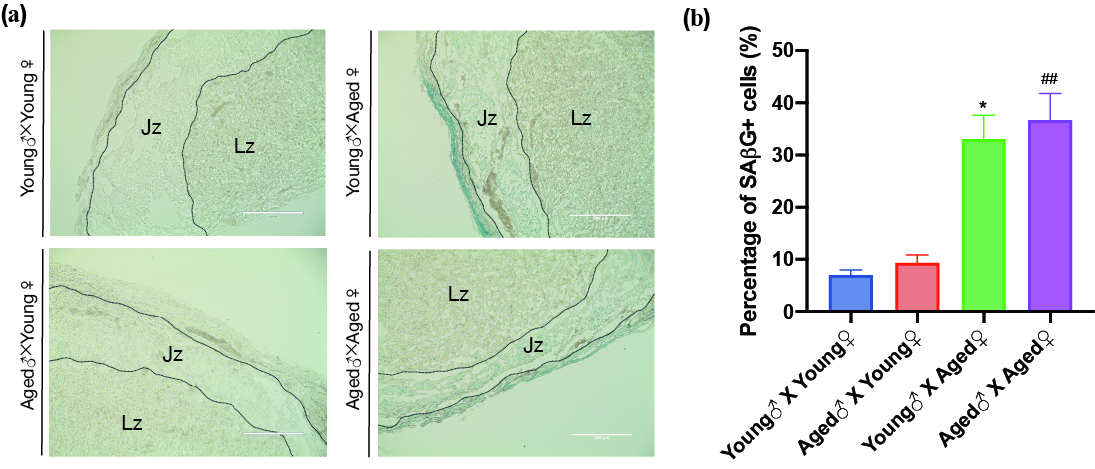


**Fig S4.** SAβG staining in mouse E18.5 placenta.

(a) Representative SAβG staining images of E18.5 mouse placentas from different mating pairs. Jz: junctional zone; Lz: labyrinth zone. Scale bar: 400 μm. AU, arbitrary unit. (b) Statistical analysis of SAβG staining. Data were analyzed using one-way ANOVA, followed by Turkey’s multiple comparison tests, *P <0.05 vs. Young♂ x Young♀ group; ##P <0.01 vs. Aged♂ x Young♀ group. All data are presented as the mean ± SEM.


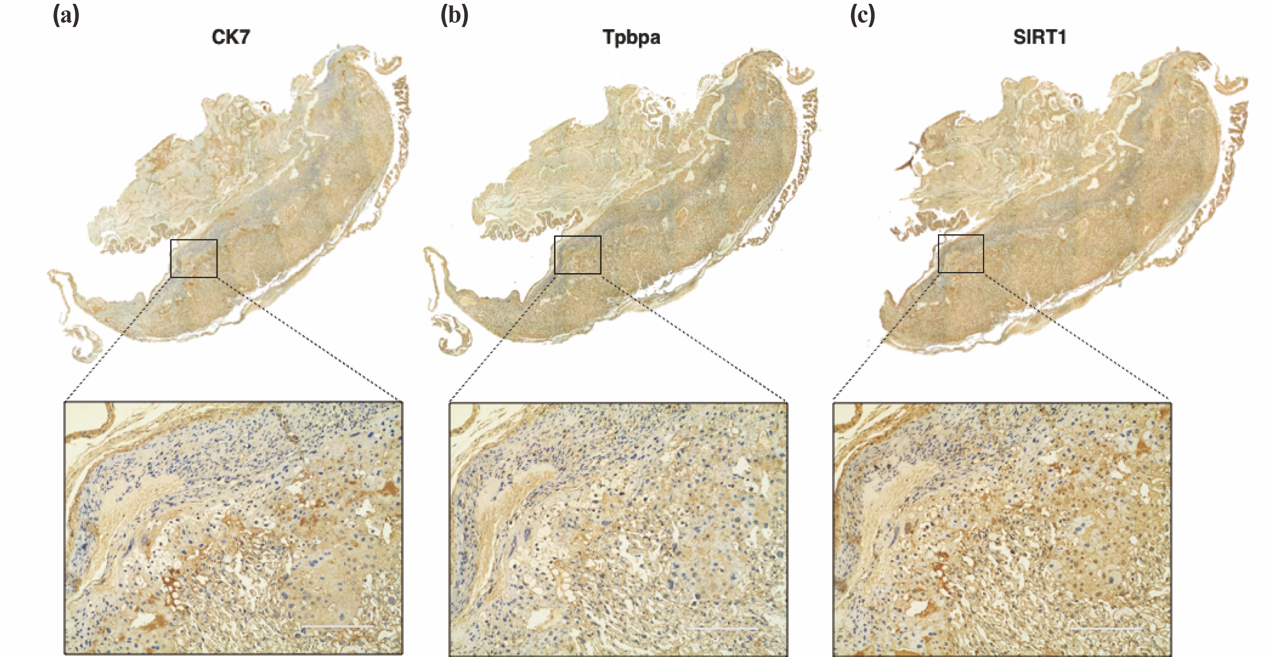


**Fig S5.** SIRT1 expression pattern in mouse placenta.

IHC staining of SIRT1 (c), CK7 (a) and Tpbpa (b) in mouse E18.5 placenta. Tpbpa is a maker of spongiotrophoblasts. Scale bar: 200 μm.


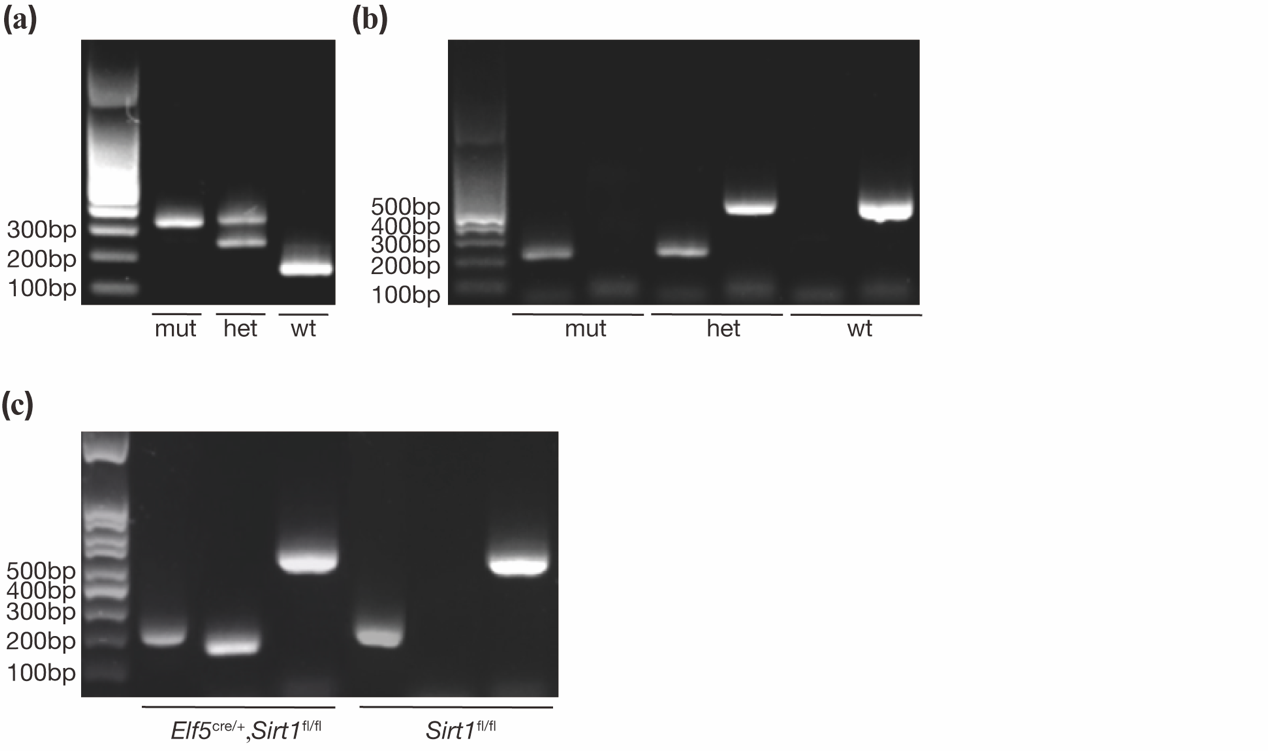


**Fig S6.** Genotyping of the transgenic mice.

(a) PCR products for the identification of *Sirt1*^loxp/loxp^ mice. The 300 bp product is the PCR product for the mutant allele, the 228 bp product is the PCR product for the wild-type allele, and the presence of both the 300 bp and 228 bp products indicates a heterozygote. (b) PCR products for the identification of *Elf5*-Cre mice. The 300 bp product is the PCR product for the mutant allele, the 500 bp is the PCR product for the the wild-type allele, and the presence of both the 300 bp and 500 bp products indicates a heterozygote. (c) PCR products for the identification of *Elf5*^cre/+^, *Sirt1*^fl/fl^ and *Sirt1*^fl/fl^ mouse placentas.

**Supplementary methods**

***In-gel tryptic digestion for LC-MS/MS***

Briefly, gel pieces were destained in 50 mM NH4HCO3 in 50% acetonitrile (v/v) until clear. Gel pieces were dehydrated with 100 μl of 100% acetonitrile for 5 min, the liquid was removed, and the gel pieces were rehydrated in 10 mM dithiothreitol and incubated at 56 °C for 60 min. The gel pieces were again dehydrated in 100% acetonitrile, the liquid was removed, and the gel pieces were rehydrated with 55 mM iodoacetamide. Samples were incubated at room temperature in the dark for 45 min. The gel pieces were washed with 50 mM NH4HCO3 and dehydrated with 100% acetonitrile. The gel pieces were rehydrated with 10 ng/μl trypsin resuspended in 50 mM NH4HCO3 on ice for 1 h. Excess liquid was removed, and the gel pieces were digested with trypsin at 37 °C overnight. Peptides were extracted with 50% acetonitrile/5% formic acid, followed by 100% acetonitrile. Peptides were dried to completion and resuspended in 2% acetonitrile/0.1% formic acid.

***LC-MS/MS***

The tryptic peptides were dissolved in 0.1% formic acid (solvent A) and directly loaded onto a homemade reversed-phase analytical column (15-cm length, 75 μm i.d.). The gradient comprised an increase from 6% to 23% solvent B (0.1% formic acid in 98% acetonitrile) over 16 min, an increase from 23% to 35% over 8 min, an increase to 80% over 3 min, and a hold at 80% for the last 3 min, all at a constant flow rate of 400 nl/min on an EASY-nLC 1000 UPLC system (ThermoFisher Scientific, Waltham, USA). The peptides were subjected to an NSI source followed by tandem mass spectrometry (MS/MS) in the Q Exactive^TM^ Plus system (Thermo Fisher Scientific, Waltham, USA) coupled online to the UPLC. The electrospray voltage applied was 2.0 kV. The m/z scan range was 350 to 1800 for a full scan, and intact peptides were detected in the Orbitrap at a resolution of 70,000. Peptides were then selected for MS/MS using an NCE setting of 28, and the fragments were detected in the Orbitrap at a resolution of 17,500. The data-dependent procedure alternated between one MS scan and 20 MS/MS scans with 15.0 s dynamic exclusion. Automatic gain control (AGC) was set at 5E4. The resulting MS/MS data were processed using Proteome Discoverer 1.3. Tandem mass spectra were searched against a database. Trypsin/P was specified as a cleavage enzyme allowing up to 2 missing cleavages. The mass error was set to 10 ppm for precursor ions and 0.02 Da for fragment ions. Carbamidomethyl groups on Cys were specified as a fixed modification, and oxidation on Met was specified as a variable modification. Peptide confidence was set at high, and the peptide ion score was set > 20.
